# Ambient-conditions spinning of functional soft fibers via engineering molecular chain networks and phase separation

**DOI:** 10.1038/s41467-023-38269-z

**Published:** 2023-06-05

**Authors:** Songlin Zhang, Mengjuan Zhou, Mingyang Liu, Zi Hao Guo, Hao Qu, Wenshuai Chen, Swee Ching Tan

**Affiliations:** 1 Department of Materials Science and Engineering, National University of Singapore, Singapore, 117574 Singapore; 2 Department of Electrical and Computer Engineering, Center for Intelligent Sensors and MEMS (CISM), NUS Graduate School for Integrative Sciences and Engineering, National University of Singapore, Singapore, 117583 Singapore; 3 Key Laboratory of Bio-based Material Science and Technology, Ministry of Education, Northeast Forestry University, 150040 Harbin, P.R. China

**Keywords:** Gels and hydrogels, Organic molecules in materials science, Polymers

## Abstract

Producing functional soft fibers via existing spinning methods is environmentally and economically costly due to the complexity of spinning equipment, involvement of copious solvents, intensive consumption of energy, and multi-step pre-/post-spinning treatments. We report a nonsolvent vapor-induced phase separation spinning approach under ambient conditions, which resembles the native spider silk fibrillation. It is enabled by the optimal rheological properties of dopes via engineering silver-coordinated molecular chain interactions and autonomous phase transition due to the nonsolvent vapor-induced phase separation effect. Fiber fibrillation under ambient conditions using a polyacrylonitrile-silver ion dope is demonstrated, along with detailed elucidations on tuning dope spinnability through rheological analysis. The obtained fibers are mechanically soft, stretchable, and electrically conductive, benefiting from elastic molecular chain networks via silver-based coordination complexes and in-situ reduced silver nanoparticles. Particularly, these fibers can be configured as wearable electronics for self-sensing and self-powering applications. Our ambient-conditions spinning approach provides a platform to create functional soft fibers with unified mechanical and electrical properties at a two-to-three order of magnitude less energy cost under ambient conditions.

## Abstract (web summary)

Producing functional soft fibers via existing spinning methods is environmentally and economically costly due to the complexity of spinning equipment, involvement of copious solvents, intensive consumption of energy. Here, the authors report a nonsolvent vapor-induced phase separation spinning approach under ambient conditions, which resembles the native spider silk fibrillation’.

## Introduction

Functional soft and stretchable fibers are indispensable for next-generation wearable electronics, such as fiber-shaped sensors, batteries, computing unit, and textile-based electronic systems^1–7^. But producing these functional soft fibers using existing spinning methods (such as electrospinning^8^, dry/wet spinning^9^, microfluidic spinning^10^, air-blow spinning^11^, or thermal drawing^12^) is cumbersome either chemically (i.e., complex in-line synthesis) or physically (i.e., specially designed equipment and multi-step pre- or post-spinning treatments). For instance, incorporating nanofillers into the spinning dope may deteriorate the dope spinnability due to the uncontrolled and unavoidable filler agglomeration. Additionally, post-treatments, including surface coating of conductive films (i.e., carbon nanotubes and silver nanowires) on soft fibers, have issues of delamination, non-stable contact, and multi-step integration process^8,13,14^. Although seamlessly integrating heterogeneous functions into the form of 1D fibers (namely, an all-in-one system) is essential for a broad range of applications^6,12,15,16^, fewer efforts have been made to address this problem via a facile and energy-efficient spinning technique^17^.

The elegance of naturally producing silks by insects under benign conditions has aroused many efforts to develop bioinspired and biomimetic approaches to fabricate synthetic fibers with different merits, especially superior mechanical properties of tensile strength^18^, toughness^19^, and damping capability^20^. Among these spinning techniques, forming functional fibers under ambient conditions—room temperature and atmospheric pressure—with combined features beyond mechanical attributes (i.e., stimuli-responsive behavior^21^, self-sensing deformations^13^, and anti-freezing^22^) is challenging. Although some favorable results have been reported on developing new spinning methods^9,16,19^, few of them are capable of producing functional fibers under ambient conditions^23–25^. These precursor dope materials are usually not drawable under ambient conditions. Moreover, additional post-treatments are necessary to endow multi-functionalities to these fibers. One critical problem is the uncontrollable spinnability of precursor elastic materials (i.e., PDMS, PU, and SEBS) due to the complicated crosslinking conditions or the inferior dope flowability^12^. Another issue is that extra processing conditions are compulsory to solidify the as-drawn precursor fibers or remove solvents, such as UV curing^26^, thermal drying^27^, coagulation bath^28^, etc.

Consequently, many disadvantages exist in current spinning techniques, including the complexity in spinning dope modification with nanofillers^29^, multi-step in-line or post-treatments^12,26^, specially-designed equipment^30^, high energy consumption^31^, and potential negative environmental impact, due to the involvement of large quantity organic/inorganic solutions^32^. In contrast, spider silks are ingeniously produced under ambient conditions that consume much less energy than most commercial synthetic fiber materials (about three orders of magnitude lower^33^). Therefore, a more sustainable and energy-efficient spinning technique to produce functional fibers under ambient conditions is in high demand, which will significantly reduce the overall CO_2_ emission due to avoiding high energy input and extra post-treatments. It is worth pointing out that spinning all kinds of “all-in-one” functional soft and stretchable fibers through an uncomplicated, energy-saving, and environmentally-friendly approach under ambient conditions is less practical in view of the current material paradigm. However, tackling this problem by probing the fiber formation mechanism of specific examples can shed light on the in-depth understanding of creating functional soft and stretchable fibers in a facile and energy-saving way—as elegant as the silk spinning by spiders or silkworms.

**Fig. 1:**
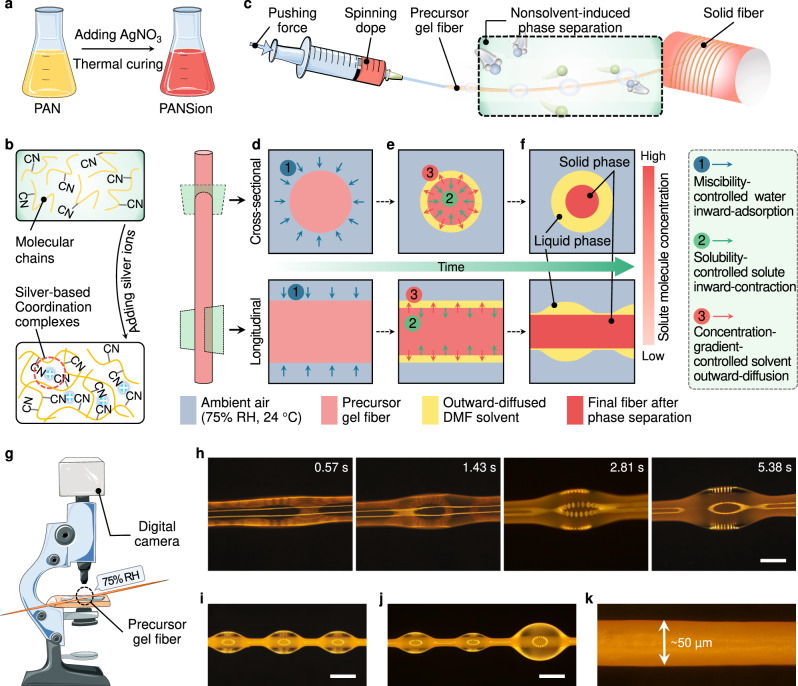
Schematic of the proposed NVIPS spinning approach. **a** Preparation of spinning dopes by introducing silver-based coordination complexes. **b** Schematic showing the difference of molecular chain networks between the control PAN and PANSion dopes. **c** Continuous fiber spinning via NVIPS spinning approach. **d**–**f** Conjectured fiber formation mechanism under ambient conditions: **d** continuous water vapor adsorption by the precursor gel fiber; **e** phase separation due to the nonsolvent (water) accumulation; **f** a solid gel fiber with solvent droplets hanging on it. **g** Optical microscope setup for observing the fiber morphology evolution under ambient conditions. **h** Image series showing the phase separation due to the NVIPS effect. **i**, **j** Solvent droplets on the solid freestanding fiber. **k** A dried PANSion fiber of ~50 µm in diameter. Components of this figure were created using Servier Medical Art templates, which are licensed under a Creative Commons Attribution 3.0 Unported License; https://smart.servier.com.

**Fig. 2:**
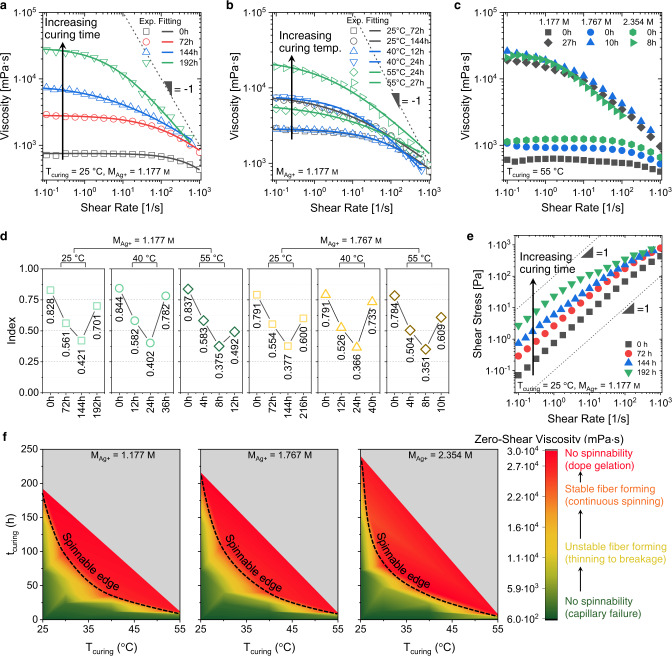
Rheological characterization of spinning dopes. **a** Viscosity changes as a function of shear rate when extending the curing time (*t*_curing_) at 25 °C. **b** Effect of the curing temperature (*T*_curing_) on the viscosity change. temp., temperature. **c** Effect of the silver ion concentration (*M*_Ag+_) on the viscosity change. **d** Evolution of the index ***n*** (extracted using the Cross-power model) for spinning dopes with different *t*_curing_, *T*_curing_, *M*_Ag+_. **e** Shear stress as a function of shear rate for spinning dopes at different *t*_curing_ at 25 °C. **f** Mapping of the spinning dopes’ viscosity at different *t*_curing_, *T*_curing_, and *M*_Ag+_. Black dashed lines marked the desired viscosity range where a good spinnability can be readily obtained.

Herein, we report a nonsolvent vapor-induced phase separation (NVIPS) spinning approach to produce functional fibers with combined mechanical and electrical attributes in a single step, surmounting problems encountered in previous spinning approaches (Fig. 1). Firstly, a polyacrylonitrile (PAN) dope (in dimethylformamide (DMF) solvent) was modified by adding silver ions (provided by silver nitrate). Consequently, the [Ag(N ≡ C − )_*x*_]^+^ coordination complexes were established between inter- and intra-chains for the mixture of PAN/silver ion solution (hereafter referred to as PANSion), resulting in an elastic polymer network (Fig. 1a, b). The optimal spinnability of PANSion dopes under ambient conditions was obtained by thermal curing at different temperatures and for various time periods, reaching the appropriate range of zero-shear viscosity (Fig. 2). Then a freestanding fiber can be readily obtained just by drawing a precursor gel thread into air [75% relative humidity (RH), 24 °C] with no additional post-spinning processes (Fig. 1c). The fiber fibrillation was triggered autonomously and instantly by the phase transition from liquid to solid due to the NVIPS effect (Fig. 1d). More importantly, the as-fabricated PANSion fibers displayed integrated heterogeneous functionalities, including good strength (1–7 MPa), high softness and stretchability (200–600% strain), and decent electrical conductivity (0.5–2 S/m). These merits enable the functional soft PANSion fibers with various potential applications as self-sensing and self-powering fiber electronics. Concisely, by engineering the molecular chain networks via establishing [Ag(N ≡ C − )_*x*_]^+^ complexes and exploiting the autonomous NVIPS effect under ambient conditions, our NVIPS spinning technique has been demonstrated to be a facile, energy-efficient, and sustainable spinning approach. It is expected to provide an alternative to current spinning methods to produce a functional soft fiber with minimum energy consumption and less volume of organic solvents.

## Results

### Fiber formation under ambient conditions via the NVIPS spinning

**Fig. 3:**
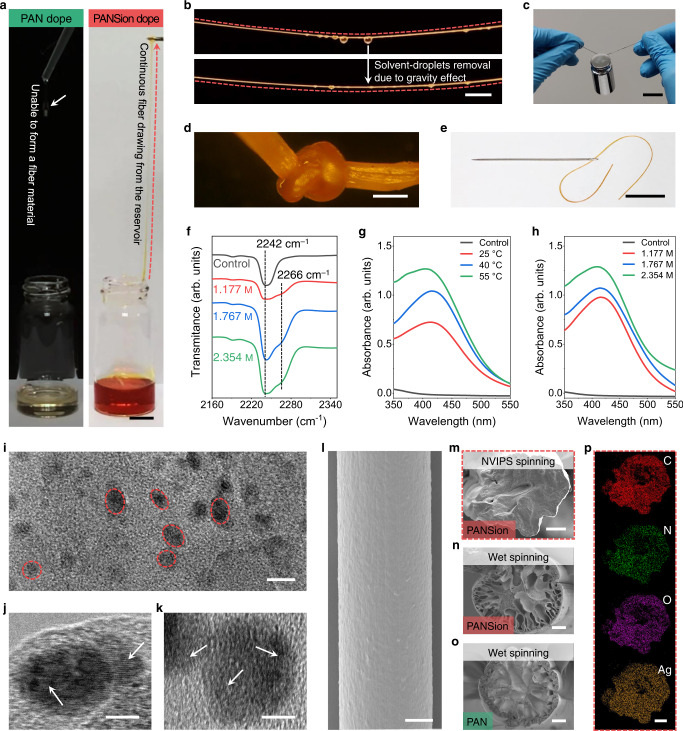
PANSion fibers via the NVIPS spinning approach. **a** Images showing the spinnability difference of PAN and PANSion dopes (scale bar, 1 cm). **b** Fiber formation via the NVIPS spinning approach where solvent droplets hanging on a fiber and can be autonomously removed due to gravity effect (scale bar, 1 cm). **c** A fiber holding a dead-load 100 g (scale bar, 1 cm). **d** An OM image of a fiber knot (scale bar, 100 µm). **e** A fiber passing through a needle hole (scale bar, 1 cm). **f** FTIR spectra of PANSion fiber with different silver ion concentrations. **g**, **h** UV-Vis spectra of spinning dopes after thermal curing at different temperatures (**g**) and silver ion concentrations (**h**). Ctrl, control. **i**–**k** TEM images showing the in-situ reduced AgNPs at both low (**i**, scale bar, 10 nm) and high (**j**, **k**, scale bar, 5 nm) magnifications. **l** SEM image showing a PANSion fiber surface. **m**–**o** Cross-sectional morphologies of PANSion fiber via NVIPS spinning approach (**m**), PANSion fiber via wet spinning approach (**n**), and control PAN fiber via wet spinning approach (**o**). **p** Elements mapping of the PANSion fiber from the red dash-line box in **m**, including C, N, O, and Ag. All scale bars for **l**–**p** are 50 µm.

To achieve an optimal spinnability at room temperature, we adjusted the molecular network structures by introducing [Ag(N ≡ C − )_*x*_]^+^ complexes to PAN solutions. In contrast to the control of PAN dopes with only Van der Waals force between neighboring chains (Fig. 1b top), PANSion dopes presented additional inter- and intra-chain interactions (i.e., entanglement and crosslinking) through [Ag(N ≡ C − )_*x*_]^+^ complexes (Fig. 1b bottom)^34–36^. This statement was first verified by the additional peak at 399.7 eV of the X-ray photoelectron spectroscopy (XPS) results, which was assigned to the [Ag(N ≡ C−)_*x*_]^+^ complexes for PANSion samples (Supplementary Fig. 1). Notably, a significant increase of the zero-shear viscosity was recorded for PANSion dopes (Fig. 2a–c), which was ascribed to the entangled and crosslinked molecular chain network via [Ag(N ≡ C−)_*x*_]^+^ complexes. Concomitantly, the unique PANSion network guaranteed the excellent ambient-condition extensibility to form a large-aspect-ratio thread (Fig. 3). In addition, the autonomous solidification of a precursor fiber was promptly triggered by the surrounding water vapor (75% RH) due to the NVIPS effect. As schemed in Fig. 1c, a PANSion fiber can be easily obtained via our NVIPS spinning approach by manually drawing or adapting a continuous extruding–stretching process (Supplementary Fig. 2 and Supplementary Movies 1 and 2). By comparison, drawing a PAN dope under ambient conditions always showed capillary failure or gravity drainage (Supplementary Fig. 3), because of the insufficient inter-/intra-chain interactions (even for a concentrated PAN dope with a comparable viscosity to PANSion dopes).

For the ambient-conditions fiber formation via NVIPS spinning, three key processes were identified from the view of both the fiber’s longitudinal section and cross section (Fig. 1d–f)^37^. Owing to the good miscibility of water and DMF, a precursor gel fiber could maintain a continuous water vapor adsorption once it was drawn into air (process ①: miscibility-controlled water inward-adsorption)^38^, eventually forming a DMF/water mixture on the fiber surface. Note that DMF/water mixture was a poor solvent for the PANSion solute^39^, thus resulting in a thin liquid layer of DMF/water on the fiber surface. Meanwhile, the PANSion solute contracted inwardly to the good solvent of DMF (process ②: solubility-controlled inward-contraction), gradually leading to a fiber diameter shrinkage with a concomitant solute concentration increase. Simultaneously, a chemical potential difference was established due to the different DMF concentrations between the fiber’s inside and surface (process ③: concentration-gradient-controlled outward-diffusion), thus expelling the continuous outward-precipitation of DMF solvent. Eventually, the solute part of PANSion contracted to a solid fiber, while the solvent part (DMF) separated from the solute and formed liquid droplets (with water) hanging on the fiber. Note that the phase transition from a liquid-like gel fiber to a solid freestanding fiber was independent and autonomous under ambient conditions (75% RH, 24 °C). Just as visualized experimentally by an optical microscope (Fig. 1g), a precursor fiber was gradually solidified with diameter shrinkage and solvent outward-precipitation (Fig. 1h and Supplementary Movie 3). The liquid phase eventually amalgamated into droplets hanging on the freestanding fiber (Fig. 1i, j). By inclining the fiber during spinning, DMF/water droplets can be easily removed or recollected due to the gravity effect (Fig. 1k, Supplementary Figs. 4 and 5, and Supplementary Movie 4).

### Tuning ambient-condition spinnability

We noticed that the zero-shear viscosity is a good indicator to assess the ambient-conditions spinnability of PANSion dopes (Supplementary Fig. 6). Having this in mind, viscosity adjustment was realized by modulating the density of [Ag(N ≡ C − )_*x*_]^+^ complexes, which, as crosslinkers, can enhance inter- and intra-chain interactions, thus effectively increasing the zero-shear viscosity to the desired level. Additionally, due to the dynamic and robust nature of [Ag(N ≡ C − )_*x*_]^+^ complexes, the PANSion dopes embraced an elastic chain network, which guaranteed the good extensibility under ambient conditions. That is to say, the entanglement/crosslinking-enhanced zero-shear viscosity via [Ag(N ≡ C − )_*x*_]^+^ complexes is the key to achieving good ambient-conditions spinnability (Supplementary Fig. 7). It is worth pointing out that a PAN dope, with either low or high concentration, is not capable of forming a long and stable precursor thread under ambient conditions either due to the high local stress or capillarity instability (Supplementary Note 1).

In our current study, the zero-shear viscosity of PANSion dopes was synergistically dictated by three factors: thermal curing time (*t*_curing_), curing temperature (*T*_curing_), and silver ion concentration (*M*_Ag+_). Thus, we characterized the zero-shear viscosity (${\eta }_{0}$) as a function of shear rate at different combinations of these parameters (Fig. 2 and Supplementary Fig. 8). For instance, when extending the curing time (*T*_curing_ = 25 °C and *M*_Ag+_ = 1.177 M), the zero-shear viscosity presented an increase by more than two orders of magnitude, from 753 to 29,573 mPa·s (Fig. 2a). Importantly, when the zero-shear viscosity was leveled up to the optimal range, in which good ambient-conditions spinnability was achieved, the shear stress presented a non-linear dependence on the shear rate (green dot in Fig. 2e and Supplementary Fig. 9). This behavior was different from the near-linear slope of non-spinnable dopes (black and red dots in Fig. 2e). In addition, the zero-shear viscosity was significantly affected by the curing temperature from 25 to 55 °C (*M*_Ag+_ = 1.177 M, and *t*_curing_ is at a comparable level), as the differences shown in Fig. 2b. For instance, to reach the optimal viscosity range of ~10^4^ mPa·s, the required curing time for dopes cured at *T*_curing_ = 40 °C was much shorter than that of dopes cured at *T*_curing_ = 25 °C. It was further shortened to just 27 h for the case of *T*_curing_ = 55 °C. Figure 2c presented the comparison of the zero-shear viscosity for dopes cured at *T*_curing_ = 55 °C while varying the curing time and silver ion concentrations. Notably, the required curing time to reach the optimal zero-shear viscosity range was decreased at a higher silver ion concentration (Supplementary Table 1).

To map the parameter zone of good ambient-condition spinnability regarding to the *t*_curing_, *T*_curing_, and *M*_Ag+_, the flow behavior of PANSion dopes was investigated. PANSion dopes only started to show a non-Newtonian behavior (i.e., shear thinning for our case) for a shear rate greater than 100 s^–1^. Once the factor of thermal curing (including *t*_curing_ and *T*_curing_) came in, the shear thinning effect became obvious at a much lower shear rate (i.e., <1 s^–1^ for *t*_curing_ = 144 and 192 h, Fig. 2a). The non-Newtonian behavior at various combinations of *t*_curing_, *T*_curing_, and *M*_Ag+_ can be explained by the flow behavior index, ***n***, using Cross power-law model^40,41^:

1 $$\eta \left(\dot{\gamma }\right)=\frac{{\eta }_{0}}{1+{\left(\frac{{\eta }_{0}\dot{\gamma }}{{\tau }^{*}}\right)}^{1-{{{{{\boldsymbol{n}}}}}}}}$$

where $\dot{\gamma }$ is the shear rate (s^–1^), τ* is the critical shear stress (Pa) (i.e., the stress at the break in the viscosity–shear rate curve), and ***n*** is the flow behavior index. Generally, a Newtonian fluid presents a value of ***n*** = 1, while a shear thinning fluid is with ***n*** < 1 and shear thickening fluid is with ***n*** > 1. By fitting Eq. (1) to the viscosity–shear rate data, we obtained the power-law index values of ***n*** under different combinations of factors, as displayed in Fig. 2d (Supplementary Figs. 8 and 10). The overall flow behavior of spinning dopes presented a decrease trend when the curing time was extended, regardless of *T*_curing_ or *M*_Ag+_. For instance, ***n*** was decreased from 0.828 to 0.421 for dopes cured at *T*_curing_ = 25 °C and *M*_Ag+_ = 1.177 M. The minimum value of ***n*** (***n***_min_) was in the range of 0.338–0.421. Particularly, the value of ***n*** showed an abrupt increase once crossing ***n***_min_ if further extending the curing time.

When ***n***_min_ was reached, the corresponding zero-shear viscosity of spinning dopes was also in the optimal range for ambient-condition spinning. Although slightly crossing the value of ***n***_min_ did not deteriorate the dope spinnability significantly, further extending the curing time should be avoided as the viscosity changed swiftly to a very high level (i.e., the gelation). Based on these discussions, we mapped the parameter zone of *t*_curing_, *T*_curing_, and *M*_Ag+_, as shown in Fig. 2f, to identify the optimal viscosity range, which was denoted by a dashed line (i.e., the spinnable viscosity edge). In other words, when the zero-shear viscosity of PANSion dopes was in the proximity of the spinnable edge, the ambient-condition spinning can be realized. Out of the spinnable edge, the dope will only result in unstable fiber formation due to insufficient entanglements or no spinnability of the gelation.

### Silver-coordinated molecular networks and in-situ reduced AgNPs

Reaping the benefits of coordination complexes-enabled good spinnability and the NVIPS effect-induced autonomous fiber fibrillation, a large aspect-ratio precursor fiber can be easily drawn (Fig. 3a), which went through phase transition instantly to a freestanding solid fiber under ambient conditions (Fig. 3b). The obtained PANSion fiber was strong (i.e., capable of holding a dead-weight 100 g, Fig. 3c), flexible and stretchable (Supplementary Fig. 11), and knottable (a knot with a radius <50 µm, Fig. 3d). This could be ascribed to the elastic molecular chain networks via [Ag(N ≡ C − )_*x*_]^+^ complexes^35^. Furthermore, the fiber diameter is variable (i.e., small enough to pass through a needle hole, Fig. 3e) by adjusting the stretching rate during spinning. Next, we verified the existence of [Ag(N ≡ C − )_*x*_]^+^ complexes (also see Supplementary Fig. 1). As the FTIR spectra results shown in Fig. 3f, a peak at 2242 cm^–1^ (−C ≡ N) was recorded for the control PAN dopes. This characteristic peak was red-shifted with an additional shoulder peak at 2266 cm^–1^ for PANSion dopes because of the chemical environment change of nitrile groups by silver ions^35^. In addition, we observed the color variations of spinning dopes after thermal curing (Supplementary Fig. 12), which was attributed to the in-situ reduced AgNPs. Previous studies reported that DMF solvent could reduce metal ions to nanoparticles, especially when the reaction temperature was elevated^42^. Indeed, we confirmed the existence of AgNPs based on UV-Vis spectra results which displayed a unique peak at 425 nm, resulting from the surface plasma resonance of AgNPs (Fig. 3g). At a higher silver ion concentration, more AgNPs were reduced in situ (Fig. 3h). Furthermore, these AgNPs, well dispersed in the spinning dopes, were directly observed by TEM images (Fig. 3i–k). Note that these in situ reduced AgNPs not only facilitated to create conductive pathways for as-prepared PANSion fibers, but also enabled the structural stabilization of the precursor gel fibers/dopes at a colloidal scale^25^.

### Mechanical properties of PANSion fibers

Although both PAN and PANSion fibers can be obtained via a wet spinning (WS) approach, the mechanical properties and morphologies of these fibers (fibers@WS) were significantly different from those via NVIPS spinning approach (fibers@NVIPS). For instance, unlike the high stretchability of PANSion fibers@NVIPS, fibers@WS were very brittle and not stretchable (Supplementary Fig. 10 and Supplementary Movie 5). Figure 3l showed the surface morphology of a PANSion fiber@NVIPS, which was smooth and different from crumped/grooved structures of fibers@WS (Supplementary Fig. 13 and 14). Moreover, the internal structures of fibers@NVIPS were solid, whereas fibers@WS were porous with many defects (Fig. 3m–o). This was due to the unstable spinodal decomposition of precursor fibers via wet spinning^43^. By contrast, the phase separation via NVIPS spinning approach was mild under ambient conditions, which facilitated the formation of a more morphology-controlled fiber. Particularly, the distribution of silver element was even in the whole cross-sectional area (Fig. 3p and Supplementary Fig. 15). These results affirmed the advantages of using NVIPS spinning approach to produce functional soft fibers over conventional spinning methods.

**Fig. 4:**
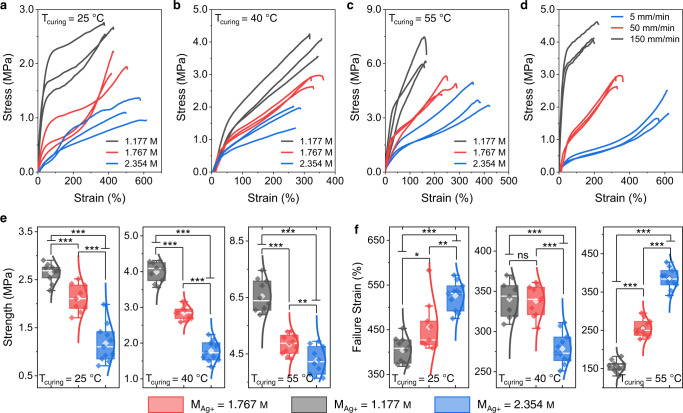
Mechanical properties of PANSion fibers. **a**–**c** Effects of silver ion concentrations on the tensile properties of PANSion fibers using spinning dopes that were thermally cured at 25 °C (**a**), 40 °C (**b**), and 55 °C (**c**). **d** The effect of the strain rate on the fiber tensile behavior. **e**, **f** Box plot (center line at the median, upper bound at 75th percentile, lower bound at 25th percentile, whiskers at the minimum and maximum values with outliers not counted; each dot represents one test, *n* = 10) of the fiber tensile strength (**e**) and failure strain (**f**). Statistics are calculated using a one-factor ANOVA test. *p* values for comparisons are shown: *p < 0.05; **p < 0.01; ***p < 0.001; ns (non-significant) *p* > 0.1.

Tensile properties of PANSion fibers were investigated as the strain-stress curves plotted in Fig. 4a–c. A pattern of the two-stage load-bearing structure was presented: a fast linear stress growth in the low-strain regime and a slow stress growth in the large strain regime. A strain “hardening” effect was recorded when raising the ramp rate from 5 to 150 mm/min (Fig. 4d). Such mechanical features were similar to typical glassy polymers^44^. Furthermore, cyclic stress-strain tests indicated a negligible residual strain when consecutively increasing strains from 0 to 150%, or cyclically stretching-releasing at 50% or 100% strains (Supplementary Fig. 16). Besides, we examined the effect of *T*_curing_ and *M*_Ag+_ on tensile behaviors of fibers. For instance, when increasing the silver ion concentrations (*T*_curing_ = 25 °C), PANSion fibers presented a tendency to decrease the tensile strength, yet an opposite trend towards increasing failure strain (Fig. 4a). This behavior pattern was also true for fibers spun from dopes cured at *T*_curing_ = 55 °C (Fig. 4c). Such mechanical behavior was possibly ascribed to the enhanced elasticity of PANSion molecular chain networks when the silver ion concentration was high. It should be noted that for fibers spun from dopes cured at *T*_curing_ = 40 °C (Fig. 4b), the failure strain presented a different behavior in contrast to fibers from dopes cured at *T*_curing_ = 25 or 55 °C. Given this, we further conducted statistical analysis on tensile strength and failure strain (Fig. 4e, f). For tensile strength, statistical significance was confirmed for all investigated fibers (Fig. 4e), with a minimum *p* < 0.01 under the one-factor ANOVA test. However, the influence of curing temperature (*T*_curing_) on the failure strain was mixed (Fig. 4f). From this perspective, future work will be conducted further to elaborate on the relationship between the parameters of preparing the spinning dopes and mechanical properties.

### Self-sensing PANSion fibers

**Fig. 5:**
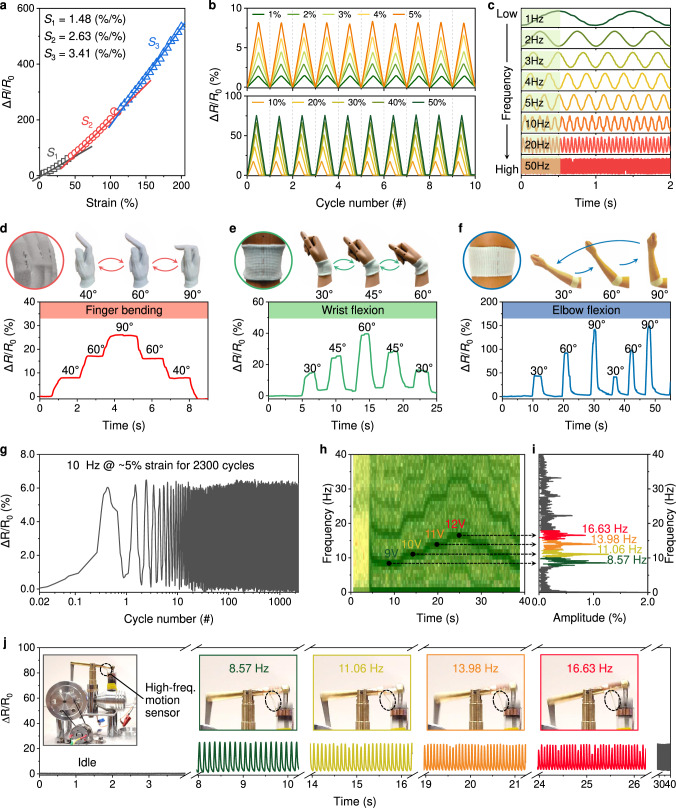
Self-sensing of PANSion fibers for body motion monitoring and high-frequency movement detection. **a** Piezoresistive effect of PANSion fibers, roughly showing three stages of sensitivity (*S*_1_, *S*_2_, and *S*_3_) in the strain range of 0–200%. **b** 10 cycles of sensing signals at both small (1–5%, top) and large (10–50%, bottom) strains. **c** Sensing signals at various motion frequencies from 1–50 Hz. **d**–**f** Human body motion monitoring using the self-sensing fiber, including a sensing glove for finger bending monitoring (**d**) and a sensitive fabric band for wrist flexion (**e**) and elbow flexion (**f**) monitoring. **g** Sensing signals under a mechanical stimulus at 10 Hz for 2300 cycles. **h**–**j** Monitoring the high-frequency movement of a converter using a self-sensing fiber: **h** the SFFT (short-time Fourier transform) analysis, **i** the corresponding frequency spectra, and **j** the sensing signals when the converter was working at various frequencies.

The piezoresistive effect was verified for a single PANSion fiber (Fig. 5) because the in situ reduced AgNPs established conductive pathways, reaching a decent electrical conductivity of 0.5–2 S/m. Note that silver ions in spinning dopes were excessive. Therefore, ionic conductivity also contributed to the overall conductivity improvement (Supplementary Fig. 17). It is worth noting that fabricating a conductive and stretchable fiber with piezoresistive sensing capability is usually associated with multi-step processes, such as coating conductive nanomaterials (i.e., carbon nanotubes) on a stretchable fiber substrate^13^, filling liquid metals into a stretchable fiber hose^12^, pre-strain engineering^45^, or conductive filler embedding^46,47^, etc. For our PANSion fibers, heterogeneous functions of both mechanical and electrical features were accomplished in a single-step process via NVIPS spinning approach, thus achieving the intrinsic self-sensing by the PANSion fiber itself. It did not require any post-spinning processes to enable the piezoresistive effect with no potential issues of coating delamination, poor adhesion, stress concentration, and electronic/mechanical property mismatch.

Given its excellent stretchability and decent conductivity, we explored the potential of PANSion fibers as self-sensing devices to monitor mechanical stimuli. Firstly, a decent sensitivity of 1.48–3.41 was achieved for strains from 0–200% (Fig. 5a). Secondly, the sensing signals were stable and repeatable at both small (1–5%) and large (10–50%) strains (Fig. 5b). As shown in Fig. 5d–f, when sewing a PANSion fiber into a fabric, a self-sensing textile was enabled to successfully detect joint motions of the human body, including finger bending and wrist/elbow flexions. More impressively, we noticed that the PANSion fiber was highly responsive to high-frequency stimuli at relatively small strains (i.e., >50 Hz at ~5–10% strain, Fig. 5c and Supplementary Fig. 18a). This feature could be credited to the resilience of fibers when strains were low. In light of the ability to detect high-frequency stimuli, we applied it to successfully monitor the working status of an electrical-mechanical converter (Fig. 5g–j). As shown in Fig. 5g, stable piezoresistive signals were presented for a mechanical stimulus at 10 Hz for ~2300 cycles. By adjusting the supplied voltage to the converter, the connecting lever (where a fiber sensor was attached, Fig. 5j) oscillated up and down at different frequencies (Supplementary Fig. 18b–d and Supplementary Movie 6). For instance, the oscillation frequency of ~8.57 Hz under a voltage of 9 V was successfully captured by the self-sensing fiber, which increased to 16.63 Hz under a 12 V voltage (Fig. 5h, i).

### Self-powered PANSion fibers

**Fig. 6:**
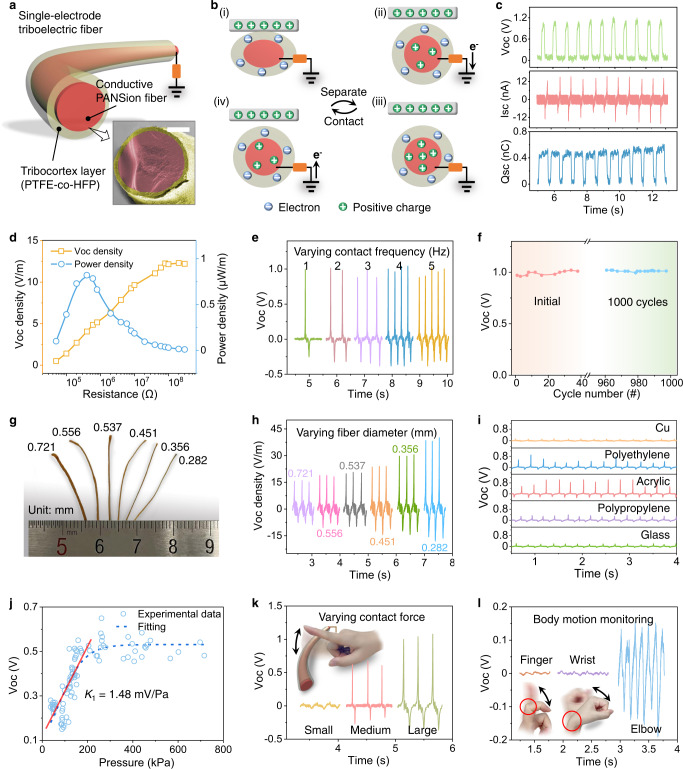
Self-powered PANSion fibers based on triboelectric mechanism. **a**, **b** Schematic diagram (**a**) and working mechanism (**b**) of the triboelectric fiber in the single-electrode mode, inside **a**: cross-section of the triboelectric fiber (Scale bar: 100 μm). **c** Electrical outputs measured at a frequency of 1.5 Hz. **d** Voltage density and power density of triboelectric fiber at a series of external loads. **e** Electrical outputs of triboelectric fiber at various frequencies (1–5 Hz). **f** Cyclic output voltage under continuous mechanical impact for 1000 cycles. **g** Digital photo of triboelectric fiber with various diameters. **h** Electrical outputs of triboelectric fibers with different diameters. **i** Output voltage corresponding to five different tapping materials. **j** Pressure sensitivity from the output voltage-pressure curve. The red line corresponds to the linear fitting. **k** The electrical signals from small, medium, and large finger tapping force. **l** The output voltage corresponding to motions from finger, wrist, and elbow for biomechanical energy harvesting.

Benefiting from the excellent softness, stretchability, and conductivity, PANSion fibers can be configured into a self-powered sensing device or a mechanical energy harvesting system based on the triboelectric mechanism (Fig. 6). As illustrated in Fig. 6a, a single-electrode triboelectric fiber was made by a sheath coating layer of poly(vinylidene fluoride-co-hexafluoropropylene) (PVDF-HFP) as the triboelectrification layer and a PANSion fiber as the core electrode (Supplementary Fig. 19). The working principle was illustrated in Fig. 6b (see details in Supplementary Note 2), which is mainly based on the coupling of triboelectrification and electrostatic induction effects^48,49^. As results shown in Fig. 6c, a 2-cm-long triboelectric fiber displayed an open-circuit voltage (*V*_OC_) of 1.2 V, short-circuit current (*I*_SC_) of 12 nA, and a short-circuit charge quantity (*Q*_SC_) of 0.5 nC. The output voltage showed an upward tendency with the increasing load resistance due to Ohm’s law (Fig. 6d). The peak instantaneous output power of the triboelectric fiber could reach 0.82 μW/m at 0.4 MΩ. It also should be noted that the internal resistance of this triboelectric fiber was much lower than those of previously reported fiber-based TENGs (Supplementary Table 2).

When varying the frequency of mechanical impact from 1–5 Hz (in the range of most human motions^50^), the triboelectric fiber presented a stable output voltage (*V*_OC_ = 1 V) and an increasing trend for short-circuit current (*I*_SC_) from 12–22 nA (Fig. 6e and Supplementary Fig. 20b). Besides, the output voltage exhibited no noticeable attenuation after 1000 cycles (Fig. 6f), indicating its good stability and durability. Interestingly, we found that a smaller diameter triboelectric fiber (i.e., from 0.28–0.72 mm, mainly determined by the size of the conductive PANSion fiber), will generate a higher output voltage (Fig. 6g, h, Supplementary Note 3, and Supplementary Fig. 20c). This effect could be ascribed to the increased electrostatic induction effect due to the considerable deformation for a small-sized triboelectric fiber when being pressed with the same external force (Supplementary Fig. 20d, e)^51^. Furthermore, when in contact with the triboelectric fiber, different materials may generate diverse electrical signals owing to the different electron affinity, which demonstrates the feasibility of the triboelectric fiber for material recognition (Fig. 6i and Supplementary Note 4). Figure 6j presented the pressure sensitivity (*K*_1_ = 1.48 mV/Pa) of the triboelectric fiber as a tactile sensor in the pressure range of less than 200 kPa, which is high enough for human motion-based pressure sensing applications (see details in Supplementary Note 5). For instance, when increasing the tapping force by a finger, the output electrical signals became noticeably higher (Fig. 6k). Taking these features into account, including superior flexibility and excellent sensing characteristics, the PANSion triboelectric fiber also could be adopted as a self-powering sensor for body motion monitoring (Fig. 6l), which will play a significant role in the wearable electronics fields.

## Discussion

Current functional soft fiber formation is usually an extrusion-dominated process and requires thermal melting of polymer blends and multi-step post-extrusion processes, which are energy-intensive. Post-treatments or filler-embedding procedures to enable electrical functions further complicate the fiber formation process and bring about potential issues. In contrast, the natural silk fibrillation by insects under ambient conditions is produced at a much higher energy efficiency level (at least three orders of magnitude less in energy intensity) yet is empowered with unique functions in-situ (i.e., directional wettability). In light of mimicking the native silk spinning, we successfully demonstrated a facile and energy-efficient way to produce functional soft PANSion fibers via a single-step spinning process under ambient conditions. Our NVIPS spinning technique under ambient conditions is enabled by surmounting two issues that are present in existing spinning methods: (1) achieving good ambient-conditions spinnability via silver-based coordination complexes and (2) accomplishing the autonomous phase transition from a precursor gel fiber to a solid freestanding fiber due to the water vapor-enabled NVIPS effect.

In summary, our functional soft PANSion fiber via NVIPS spinning approach does not require any post-spinning treatments, such as thermal heating, in-line UV curing, coagulation bath, nor exposure to hot steam. The required energy to spin PANSion fibers can be further reduced via a manual dry-spinning way. According to a recent study by Cruz et al.^23^, the energy required for our NVIPS spinning approach is estimated to be roughly two orders of magnitude less than commercial synthetic fibers (only shearing input is needed when using an extrusion process, Fig. 1c). More importantly, electrical functionality is simultaneously obtained for PANSion fibers along with mechanical softness and stretchability. The merits of extraordinary energy efficiency and seamless mechanical-electrical function integration via a single-step process represent significant progress in producing functional soft fibers under ambient conditions. As a proof-of-concept, PANSion fibers were successfully tested for various applications because of the unified mechanical-electrical properties, including self-sensing fiber electronics to monitor mechanical stimuli and self-powering for sensing and energy harvesting. In short, the formation of PANSion fibers via our proposed NVIPS spinning approach resembles—to some extent—the natural silk fibrillation by spiders. These results of recreating the spider silk fibrillation process under ambient conditions provide insight into developing facile and energy-saving spinning technologies to afford a more sustainable future with abundant functional soft fibers.

## Methods

### Materials

Polyacrylonitrile (PAN, >99%, Mw 150,000), *N*,*N*-dimethylformamide (DMF, 99.8%), silver nitrate (AgNO_3_, ACS reagent, ≥99.0%), and poly(vinylidene fluoride-co-hexafluoropropylene) (PVDF-co-HFP) were purchased from Sigma-Aldrich. All chemicals were used as received without any post-treatment.

### Preparation of spinning dopes

PAN/DMF solutions were firstly prepared by just mixing the PAN powder and DMF solvent under magnetic stirring. Then AgNO_3_ salt was added to the PAN/DMF solutions to obtain the as-prepared PANSion dopes (see Supplementary Table 1 for detailed dope compositions). Then thermal curing for a specific time at 25, 40, and 55 °C was applied to enable a good ambient-condition spinnability for PANSion dopes. The same procedures were applied to prepare the control PAN dopes which were just pure PAN/DMF solutions.

### Spinning PANSion fibers via the NVIPS spinning approach

Spinnable PANSion dopes can be used for PANSion fiber preparation under ambient conditions, either via a manual spinning way or using a syringe-based extrusion system (Supplementary Fig. 1d). Typically, for manual spinning, one can obtain a fiber by stretching a gel ball of PANSion dopes (75% RH at 24 °C). Fiber diameter can be adjusted by controlling the stretching ratio. For continuous spinning, extruding a dope-filled syringe can result in fiber formation while continuously stretching and collecting the fiber on a bobbin. Fibers could be drawn continuously and uniformly with a length longer than two meters. Note that the PAN dopes are not suitable for spinning via NVIPS spinning approach. However, both as-prepared PANSion and PAN dopes can be used for producing fibers via a wet spinning approach (Supplementary Fig. 1c).

### Self-sensing and self-powered fiber devices

PANSion fibers were used as self-sensing strain sensors without any post treatments. They can be further integrated with commercial textiles, thus enabling sensitive wearables electronics. In this study, body motions of elbow and wrist flexion and finger bending were monitored by the sensitive textiles or gloves. Resistance changes of the self-sensing fiber sensors during the body motions were recorded by a multimeter (Keithley 6500). For self-powered sensing devices, a PANSion fiber as the electrode was uniformly coated with a PVDF-HFP layer and then dried at room temperature.

### Characterization

Rheological properties of spinning dopes were characterized using a Rheometer (Anton Paar, MCR 302) with a parallel plate of ∅25 mm in the shear rate range of 0.1 to 1000 1/s. A tensile test was performed on a universal mechanical testing machine at 50 mm/min. For cyclic tests, the fiber was first stretched to a designed strain and then rested for intervals of 0, 5, 10, 20, and 30 min before the next stretching. UV-Vis (ultraviolet–visible) spectra were recorded from 800 to 250 nm using an Agilent Cary 5000. FTIR (Fourier-transform infrared) spectra were recorded using a Thermo Nicolet NEXUS 470 spectrometer with a wavenumber from 4000 cm^−1^ to 400 cm^−1^. AgNPs were characterized using Transmission electron microscopy (TEM, JEOL JEM 2010F). Field-emission scanning electron microscopy (FE-SEM, Zeiss Supra 300) with an accelerating voltage at 3 kV was used for fiber morphology characterization. X-ray photoelectron spectroscopy (XPS, Kratos Analytical, Axis Ultra DLD) was used to analyze the chemical changes induced by the formation of silver-based coordination complexes. Optical images were recorded using an optical microscope (OM), Nikon Eclipse LV1000, equipped with Digital Sight DS-U1. Resistance changes (Δ*R*/*R*_0_) of fibers when subjected to various strains were collected using a multimeter (Keithley 6500). Sensing data from 1 to 50 Hz were first collected using a linear motor as the frequency generator. The electric output performances of the self-powered PANSion fiber were characterized by a Keithley 6514 electrometer. This work has been obtained informed consent from all participants and we have complied with all relevant ethical regulations.

## Supplementary information

Supplementary Information

Description of Additional Supplementary Files

Supplementary Movie 1

Supplementary Movie 2

Supplementary Movie 3

Supplementary Movie 4

Supplementary Movie 5

Supplementary Movie 6

## Data Availability

All the data supporting the findings in this study are available in the article and Supplementary information files or available from the corresponding authors upon request.
