# Supplementary material for: Ambient-conditions spinning of functional soft fibers via engineering molecular chain networks and phase separation
Source: Nat Commun. 2023 Jun 5;14:3245. doi: 10.1038/s41467-023-38269-z (PMC10241934; doi:10.1038/s41467-023-38269-z)
Supplement: Supplementary file 1 — Supplementary Information [file 41467_2023_38269_MOESM1_ESM.pdf]

Supplementary Information for

Ambient-conditions spinning of functional soft fibers via engineering molecular chain networks and phase separation

Songlin Zhang[1], Mengjuan Zhou[1#], Mingyang Liu[1#], Zi Hao Guo[2], Hao Qu[1], Wenshuai Chen[3], Swee Ching Tan[1]*

[1]Department of Materials Science and Engineering, National University of Singapore, Singapore, 117574, Singapore

[2]Department of Electrical and Computer Engineering, Center for Intelligent Sensors and MEMS (CISM), NUS Graduate School for Integrative Sciences and Engineering, National University of Singapore, 117583, Singapore

[3]Key Laboratory of Bio-based Material Science and Technology, Ministry of Education, Northeast Forestry University, Harbin 150040, P.R. China

[#]These authors contributed equally

[*]To whom correspondence should be addressed: S.W.T (msetansc@nus.edu.sg)

Supplementary Note 1: NVIPS spinning under ambient conditions

Note that the successful fiber formation under ambient conditions via our proposed NVIPS spinning was influenced by many other factors which are not specifically discussed in the current study due to the article length limitation. For instance, the humidity level is important to initiate the precursor gel fiber phase separation. The fiber spinning demonstration in this study was carried out at 75% RH. However, when the humidity level is too low (i.e., <25% RH), the phase separation was not triggered even after leaving the precursor gel for 5 hours. Additionally, a precursor fiber with a larger diameter would take a longer time to reach the end of the phase separation compared to the one with a smaller diameter. Further studies are needed to fully elaborate the fiber formation via the NVIPS spinning under ambient conditions. Regarding to the selection of solvent when preparing the spinning dopes, there are a few requirements, including (1) being capable of solvating the silver salt and polymer, (2) no coordination bond with the silver ions, (3) being capable of partially reducing the silver ions to silver nanoparticles, (4) being miscible with water, etc. Definitely, more work will be needed to fully elaborate the fiber formation under ambient conditions.

Supplementary Note 2: Self-powered PANSion fiber triboelectric nanogenerator

1. Fiber TENG preparation and performance characterization.

PVDF-HFP was employed as the triboelectric material owing to its high electronegativity, exceptional charge density, and excellent chemical and mechanical stabilities[1-3]. PVDF-HFP precursor solution (acetone as the solvent) was facilely dip-coated onto the surface of a conductive PANSion fiber, which was subsequently air-dried overnight. Eventually, PVDF-HFP was successfully and uniformly deposited on the fiber substrate through the combination of strong electrostatic attraction and Van der Waals force[4,5].

A measurement platform (a single-electrode mode) is built as shown in **Supplementary Fig. 20a**. The triboelectric fiber with a length of 2 cm is attached to the surface of the PTFE substrate, and a piece of an acrylic panel with a length of 2 cm as the friction material is fixed to the vibrating surface of a linear motor. The acrylic panel continuously contacts with and separates from the triboelectric fiber under a frequency of 1.5 Hz and an applied force of 3 N. The output power density is calculated by the equation of $W=U^2/(RL)$, where U, R, and L are the output voltage, load resistance, and the effective area, respectively. For motion monitoring, a triboelectric fiber is mounted on the finger, wrist, and elbow. As the joints bend or move, the electrical signals are generated periodically owing to the contact and separation between the sensor and the human skin.

2. Working mechanism of the single-electrode triboelectric fiber

When the triboelectric fiber is touched with external tribomaterials (acrylic panel or skin), electrons are transferred from the tribomaterials to the fiber, owing to the high electron affinity of the PVDF-HFP sheath, and there are positive charges correspondingly accumulating on the tribomaterials (**Stage i**). Then, upon the separation between them, an electrical potential difference is generated, inducing the positive charges

on the fiber electrode, which is accompanied by free electrons flowing from the conductive PANSion fiber to the ground (**Stage ii)**. Finally, there is an electrostatic equilibrium once the two materials are separated completely (**Stage iii**). Next, when the external tribomaterials approach the triboelectric fiber again, electrons flow back to the PANSion fiber from the ground, leading to a reversed electrical signal (**Stage iv**). Thus, the alternating electricity output is generated continuously through contact and separation process.

3. Effect of fiber diameter on the output performance

As displayed in Fig. 6g and h, the output voltage was inversely proportional to the fiber diameter ranging from 0.28 to 0.72 mm, in which the size of diameter was determined by the thickness of the core conductive PANSion fiber. As the fiber diameter increased from 0.28 mm to 0.72 mm, the output voltage density first decreased rapidly from 38 V/m to 19 V/m, and then the reduction flattened out, from 19 V/m to 15 V/m. When the fiber diameter is in the range of 0.28-0.72 mm, the generated electrical signals show an inverse trend with the fiber diameter. The reason was that under the same external force, fiber with a small diameter was applied with a high pressure, resulting in a large deformation (**Supplementary Fig. 20d**), which induced more tribo-charges owing to the effective synergism of triboelectrification and electrostatic induction. Comparatively, triboelectric fiber with a large diameter exhibited a small deformation, showing a low output voltage due to the decrease of electrostatic induction effect[6].

4. The capability of material recognition

Five common materials, i.e., copper foil, polyethylene, acrylic, polypropylene, and glass, were used to contact and separate with the fiber TENG under the same frequency and force, as shown in Fig. 6i. The generated voltages were 0.16 V, 0.5 V, 0.98 V, 0.36 V, and 0.2 V, respectively. The reason was that different materials possess discrepant electron affinity potential energies[7]. Thus, once the fiber TENG contacted with them, differentiated electrostatic charge generation was induced between them. Results demonstrated that the triboelectric fiber is promising as a sensor to identify materials. Definitely, recognition accuracy can be significantly improved by applying intelligent algorithms which we will consider in the future.

5. Pressure sensitivity

The pressure sensitivity of the triboelectric fiber as a tactile sensor is studied through exploring the relationship between the electrical output signal and the load pressure (Fig. 6j) under high humidity condition (70 %RH). The generated V_{oc} shows an upward trend with the increase of the loading pressure to 200 kPa, and then it reaches a plateau. The tactile sensor presents a sensitivity of 1.48 mV/Pa. The reasons were that with the increase of loading pressure, the deformation of the fiber TENG was more and more obvious, so that the contact area between it and external friction material increased, resulting in an increase of the output signal. When the loading pressure was high enough, the triboelectric fiber fully contacted with the friction material, without further change of deformation, eventually leading to a saturation of its output voltage.

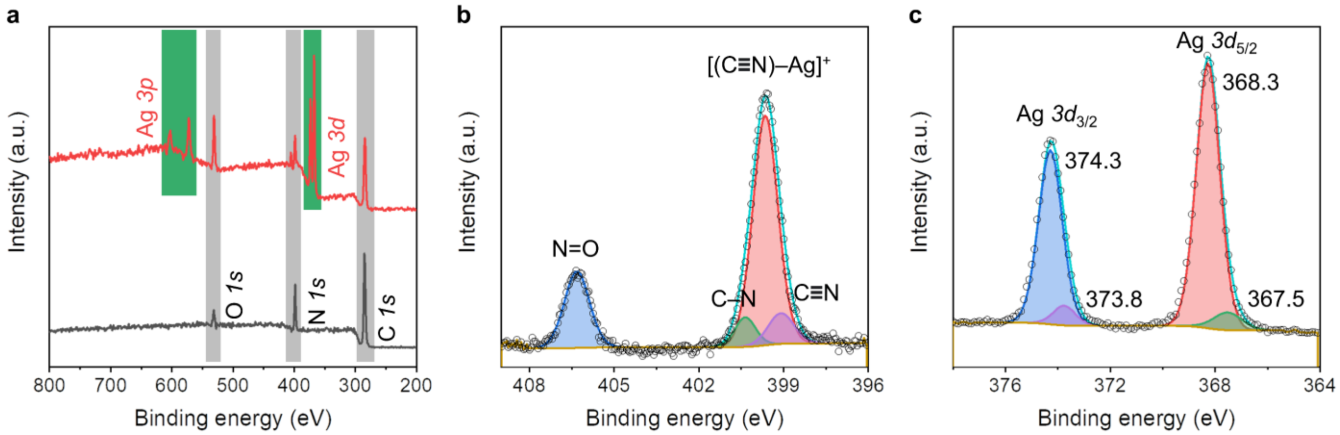

Supplementary Figure 1. XPS results of both PAN and PANSion samples. a, XPS survey results. **b**–**c**, High-resolution peaks of N 1s for PANSion (b) and PAN (c). Note that the additional peak at 399.7 eV for PANSion sample was assigned to the $[Ag(N{\equiv}C-)_x]^+$ complexes, which was estimated to be ca. 7.25 times of the C≡N in terms of concentration (%). Additionally, for the high-resolution peaks of Ag 3d (c), both Ag(0) and Ag(+) were presented, which was estimated to be ca. 9: 1 [Ag(0): Ag(+)].

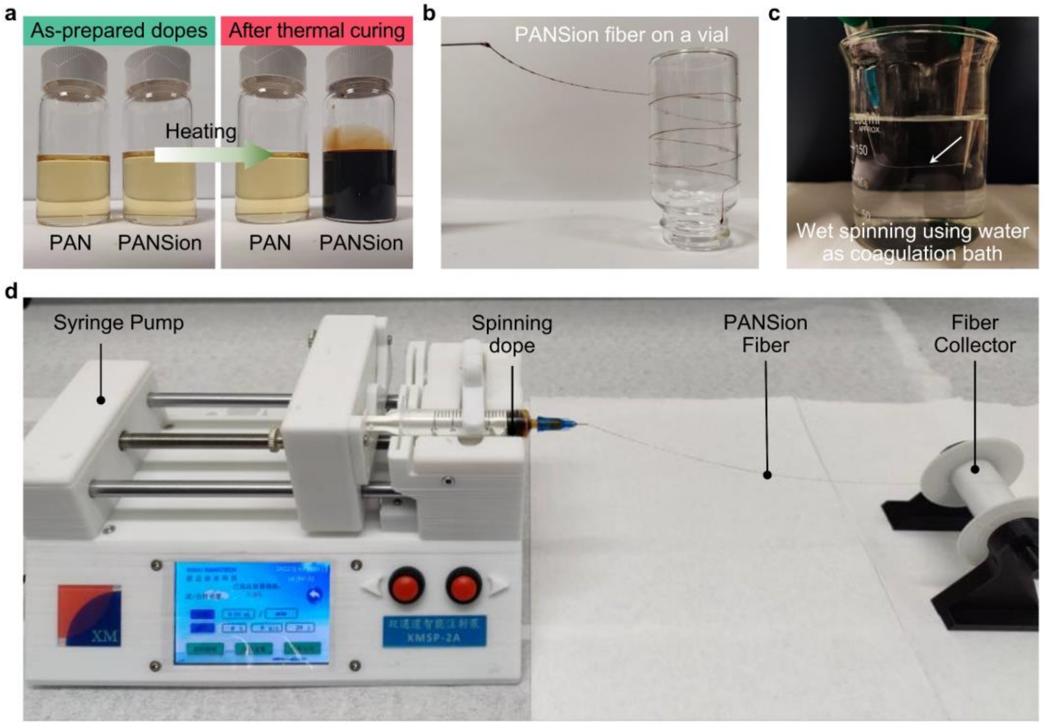

Supplementary Figure 2. Spinning dopes and the fiber spinning via wet spinning approach and our proposed NVIPS spinning approach. a, Spinning dopes before and after thermal curing. **b**, Continuously spinning a PANSion fiber on a vial. **c**, Wet spinning using a water solution as a coagulation bath. **d**, Setup of the extrusion-based continuous spinning to produce PANSion fiber via NVIPS spinning approach.

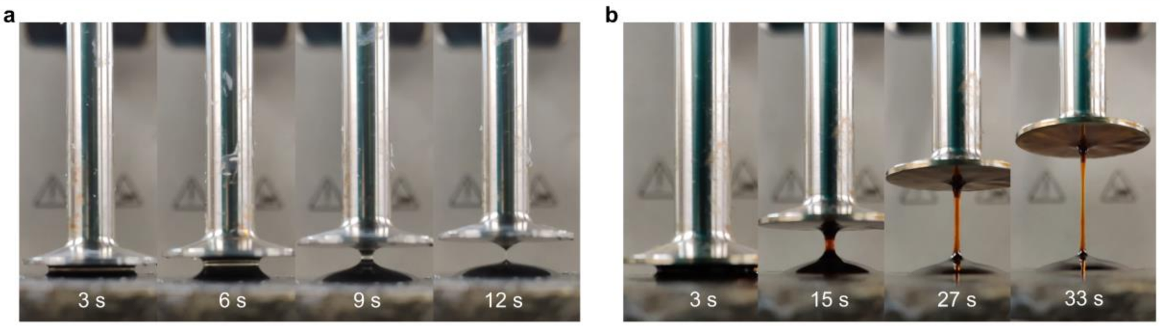

Supplementary Figure 3. Spinnability comparison between PAN and PANSion dopes by mechanical extending. **a**, PAN dope. **b**, PANSion dope. Spinnability (or extensibility) difference between the two dopes resulted from the molecular chain network difference. For PANSion dopes, additional inter- and intra-chain interactions were established by introducing silver-based coordination complexes, $[Ag(N{\equiv}C-)_x]^+$.

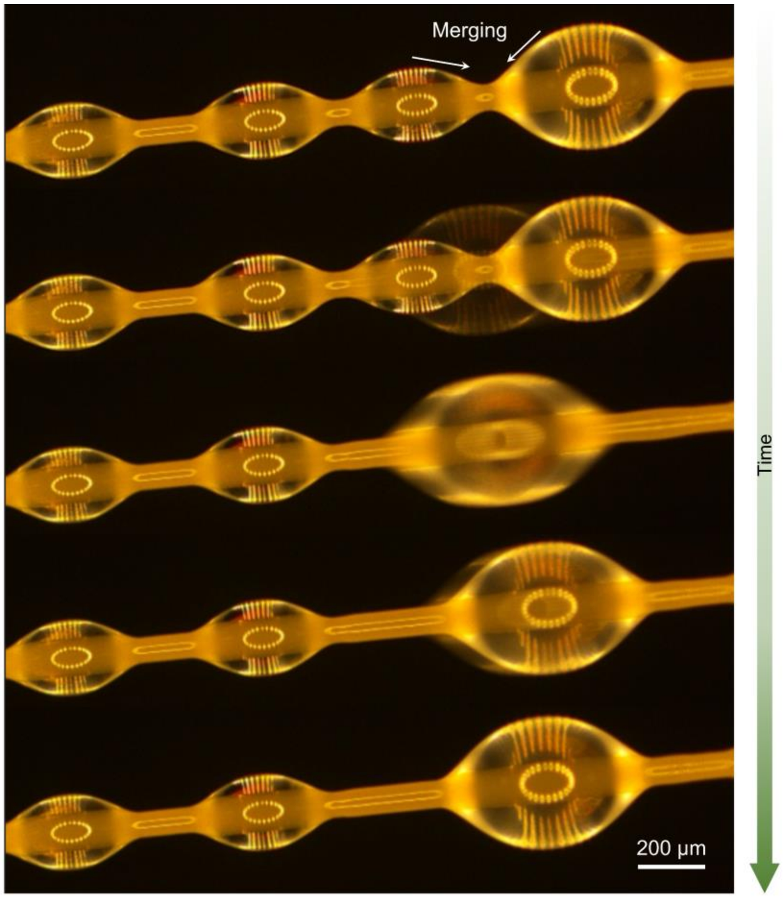

Supplementary Figure 4. Solvent droplets merged to each other due to the surface tension effect.

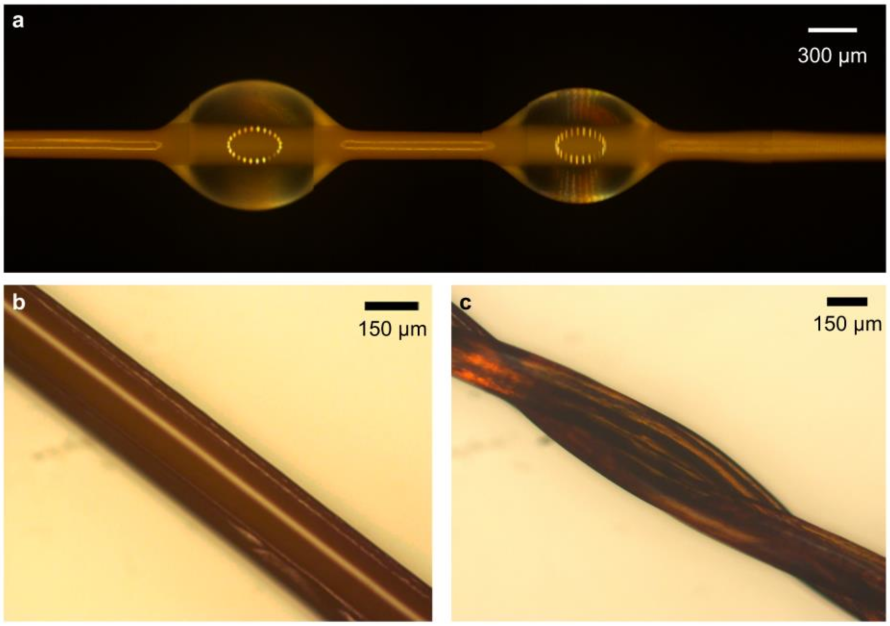

Supplementary Figure 5. OM images showing the as-spun PANSion fiber and the dry PANSion fiber. a, A PANSion fiber with two solvent droplets hanging on it. **b**, A dry PANSion fiber. **c**, A twisted yarn with two PANSion fibers.

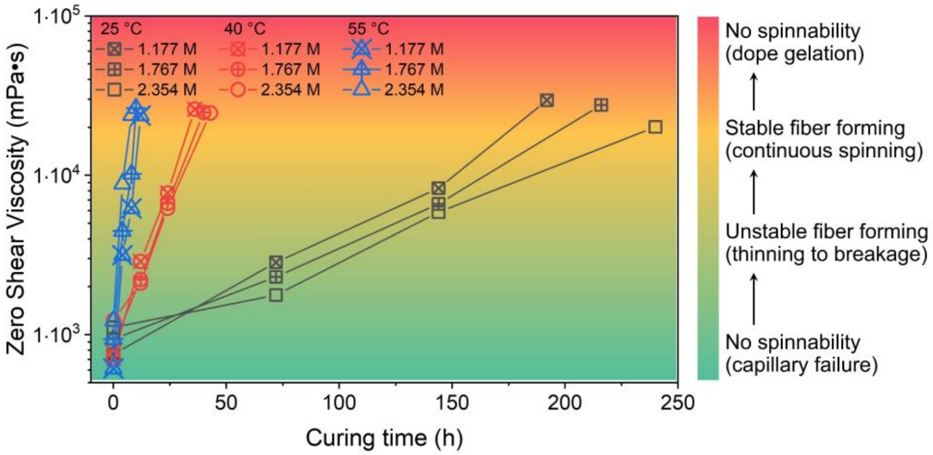

Supplementary Figure 6. Viscosity changes of various spinning dopes when varying the parameters of thermal curing time, curing temperature, and silver ion concentrations. The viscosity of spinning dopes was continuously increased when extending the curing time regardless of the curing temperature or silver ion concentration. Concomitantly, the spinnability was also improved from no spinnability to unstable fiber formation, stable fiber formation and no spinnability again due to the dope gelation.

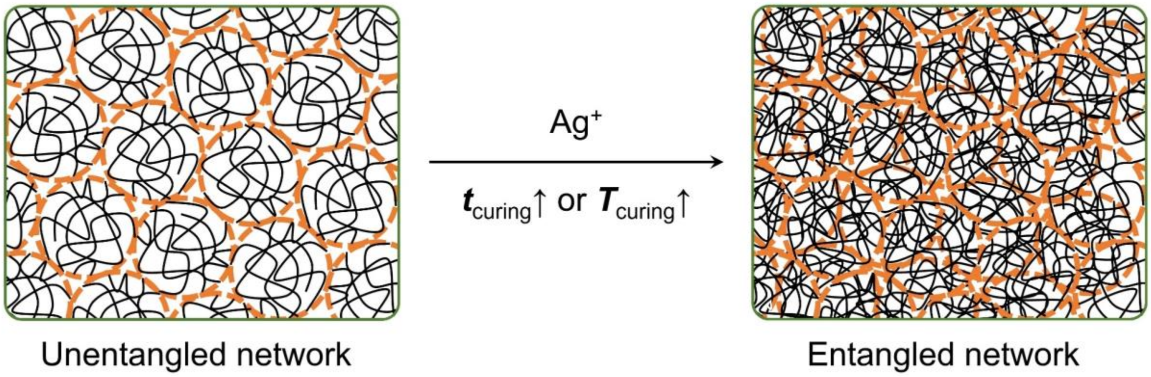

Supplementary Figure 7. Schematic showing the network structure difference between PAN and PANSion dopes. After introducing the silver-based coordination complexes, the increased viscosity of PANSion dopes resulted from the additional molecular chain interactions, leading to an entangled and crosslinked network structure that is highly elastic. In contrast, PAN dopes (even at a very high concentration, >20 wt%) presented only Van der Waals force between neighboring molecular chains, which usually displayed capillary failure when subject to mechanical stretching.

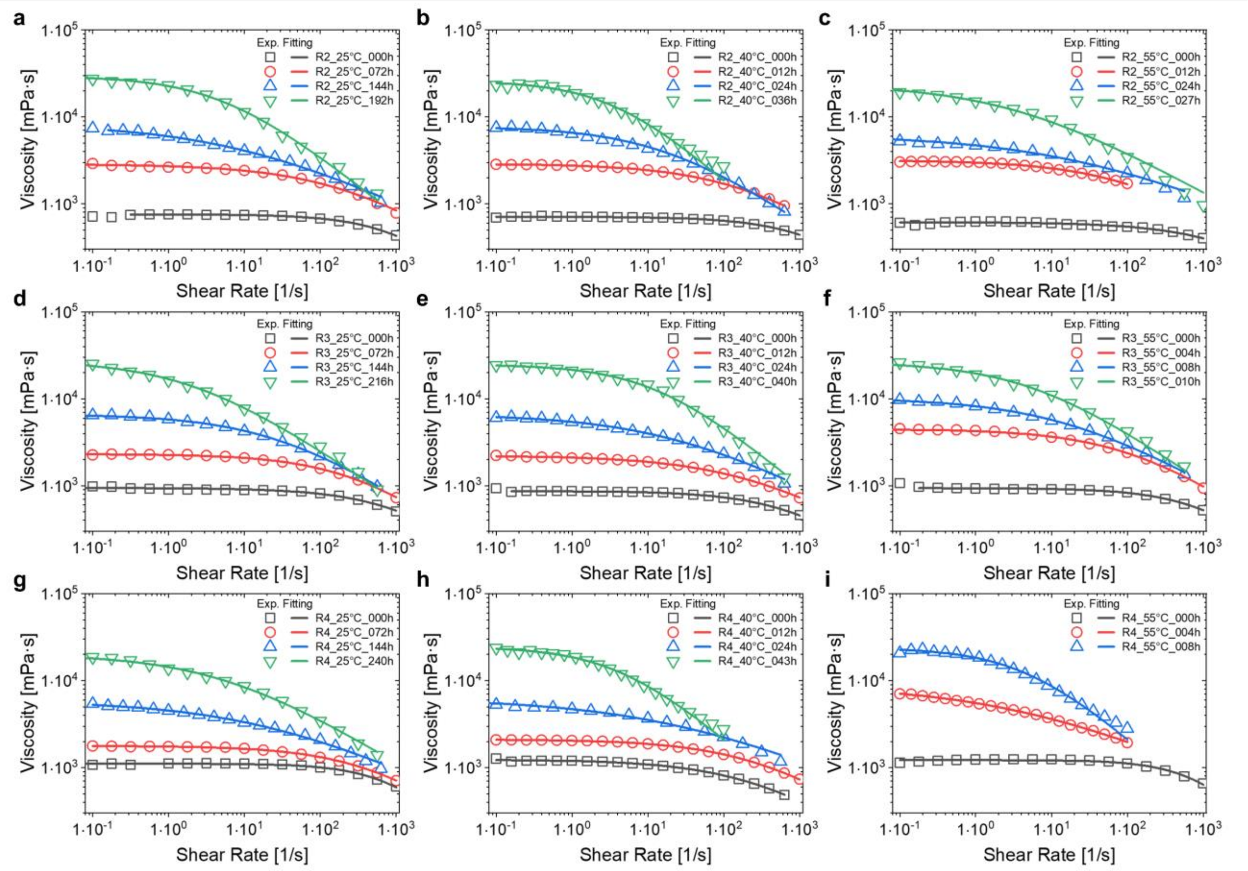

Supplementary Figure 8. Viscosity evolution of PANSion dopes when varying the curing time, curing temperature, and silver ion concentrations. a–c, PANSion dopes with M_{Ag+} = 1.177 M (R2). **d–f**, PANSion dopes with M_{Ag+} = 1.767 M (R3). **g–i**, PANSion dopes with M_{Ag+} = 2.354 (R4).

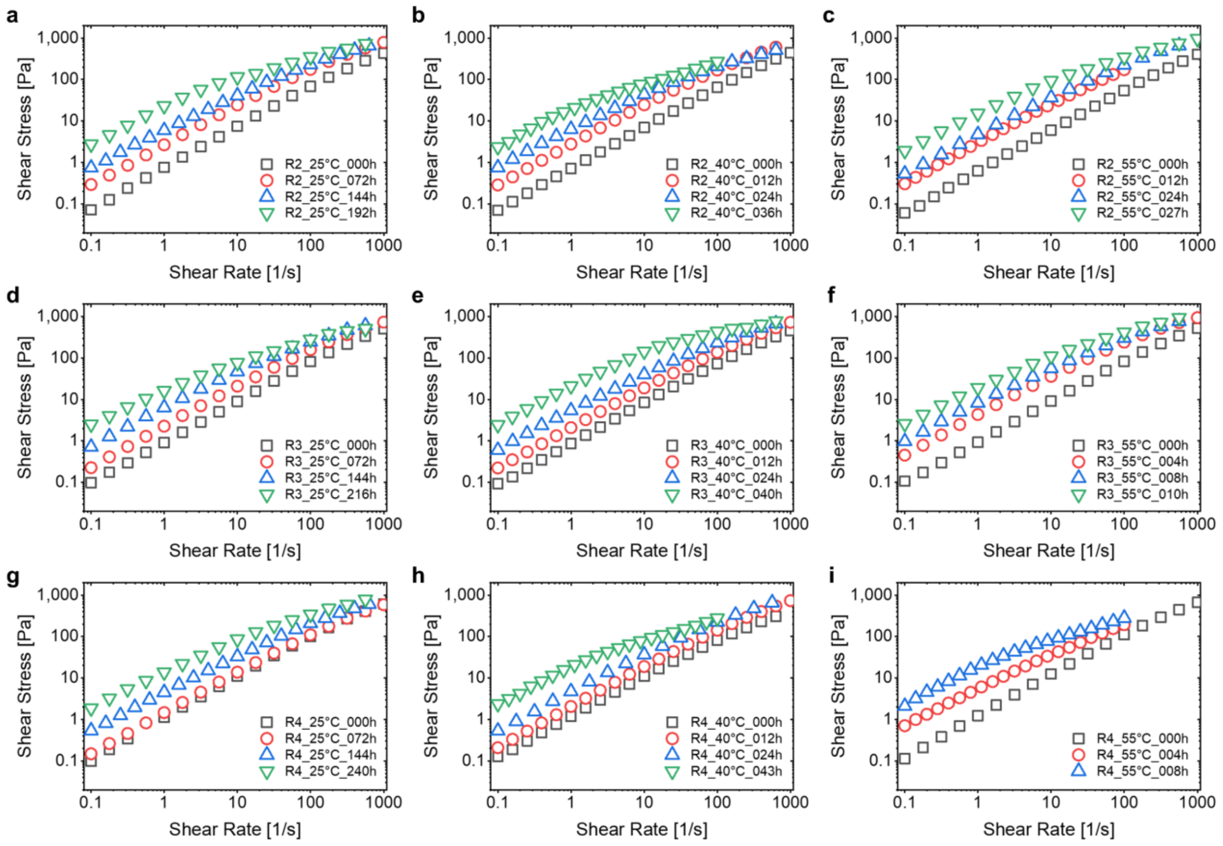

Supplementary Figure 9. Shear stress as a function of shear rate for PANSion dopes. a–c, PANSion dopes with M_{Ag+} = 1.177 M (R2). **d–f**, PANSion dopes with M_{Ag+} = 1.767 M (R3). **g–i**, PANSion dopes with M_{Ag+} = 2.354 M (R4).

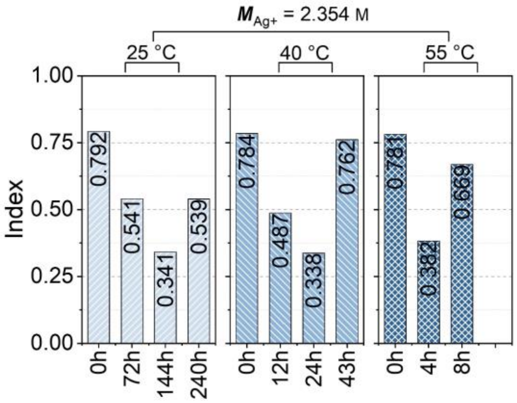

Supplementary Figure 10. Evolution of the index *n* (extracted using the Cross-power model) for spinning dopes with M_{Ag+} = 2.354 M.

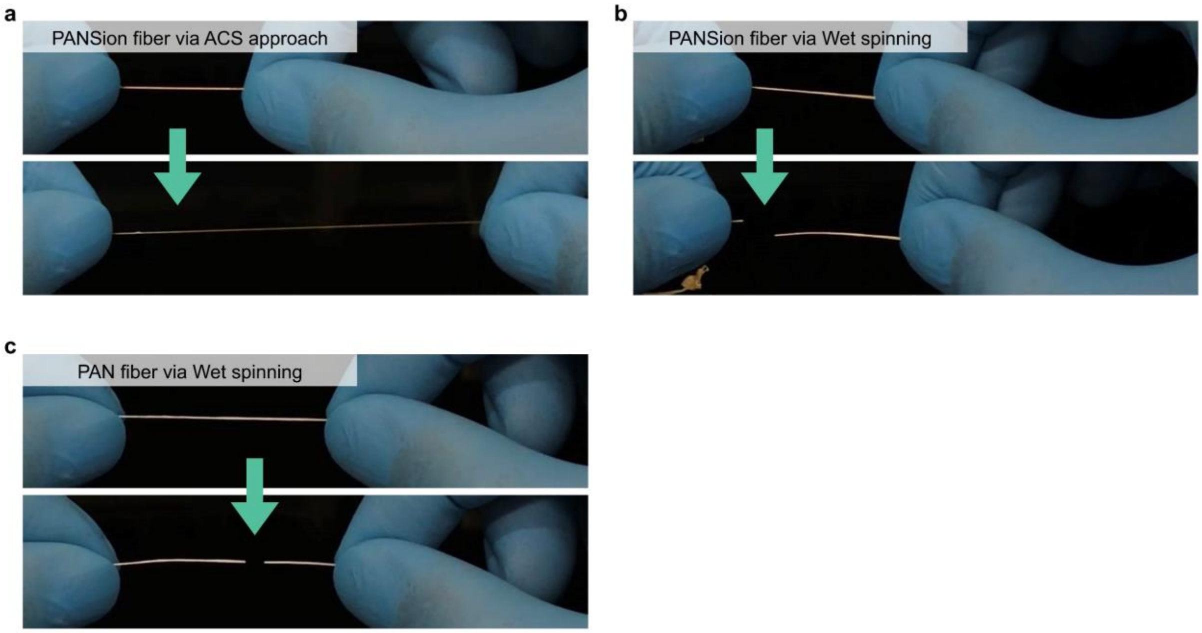

Supplementary Figure 11. Stretchability comparison between fibers prepared via NVIPS spinning approach and wet spinning approach. a, PANSion fibers via NVIPS spinning approach. **b**, PANSion fibers via wet spinning approach. **c**, PAN fibers via wet spinning approach. Different spinning conditions resulted in the different structures of the corresponding fibers. Eventually, the fibers via NVIPS spinning and wet spinning approaches presented different mechanical properties. For instance, PANSion fibers@NVIPS were highly stretchable. However, PANSion fibers@WS were not stretchable at all. PAN fibers@WS were very brittle. Therefore, our NVIPS spinning approach is superior to wet spinning to produce functional soft fibers.

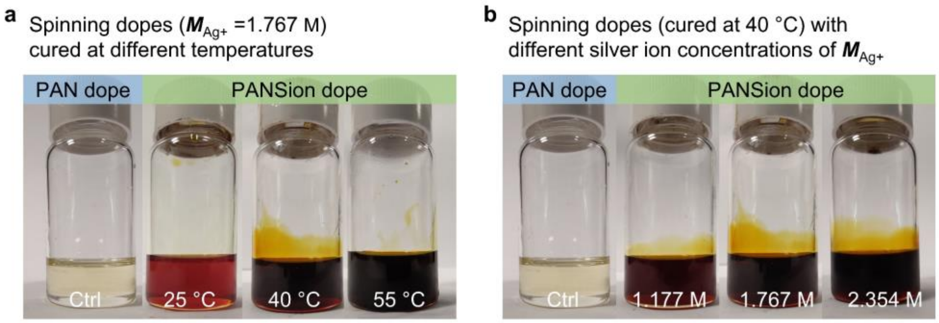

Supplementary Figure 12. Digital images showing the color differences of various spinning dopes. **a**, Spinning dopes cured at different temperatures while silver ion concentration was fixed. **b**, Spinning dopes with different silver ion concentrations while the curing temperature was fixed. The color difference was due to the in-situ reduced AgNPs, which were in different concentrations when the curing temperature or silver ion concentration varied.

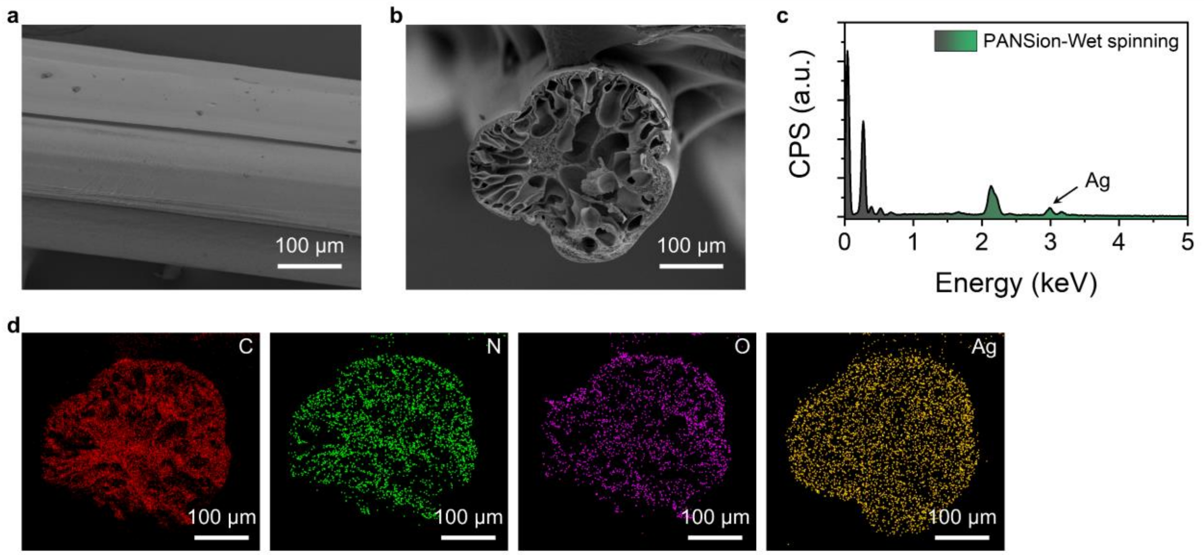

Supplementary Figure 13. Morphologies and element analysis of PANSion fibers via wet spinning approach (PANSion fibers@WS). a, Surface morphology of a PANSion fiber@WS. **b**, Cross-sectional morphology with many internal pores. **c–d**, EDX spectrum (c) and element mapping (d).

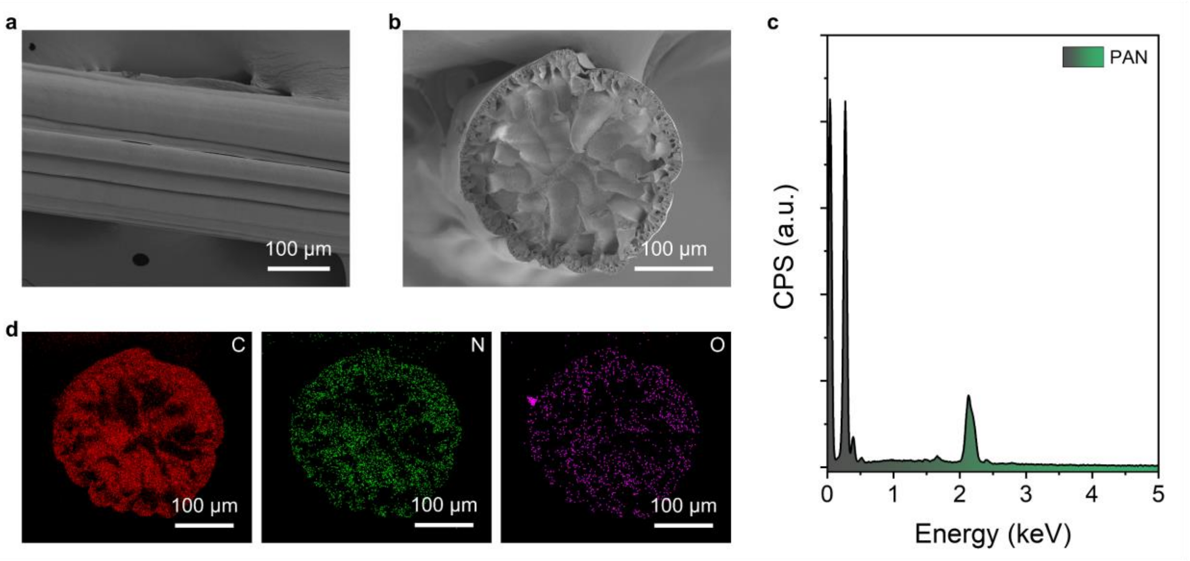

Supplementary Figure 14. Morphologies and element analysis of PAN fibers via wet spinning approach (PAN fibers@WS). a, Surface morphology of a PANSion fiber@WS. **b**, Cross-sectional morphology with many internal pores. **c–d**, EDX spectrum (c) and element mapping (d).

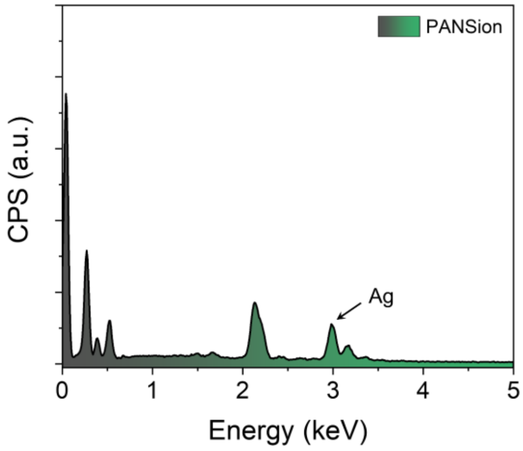

Supplementary Figure 15. EDX spectrum of PANSion fibers@NVIPS.

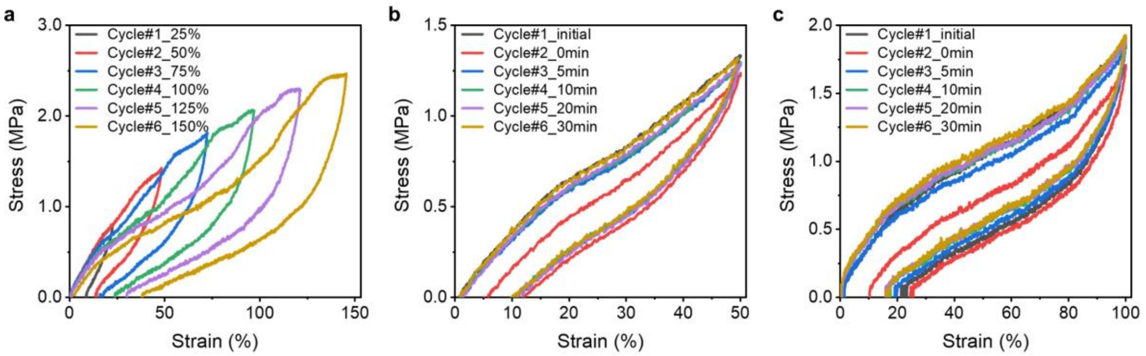

Supplementary Figure 16. Cyclic stretching-releasing of PANSion fibers at different strains. a, Consecutive stretching-releasing tests on a PANSion fiber while increasing the applied strain from 25% to 150% (interval relaxation time: 10 min). **b–c**, Cyclic stretching-releasing tests on a PANSion fiber with different interval relaxation times at a fixed strain: (b) 50% and (c) 100%.

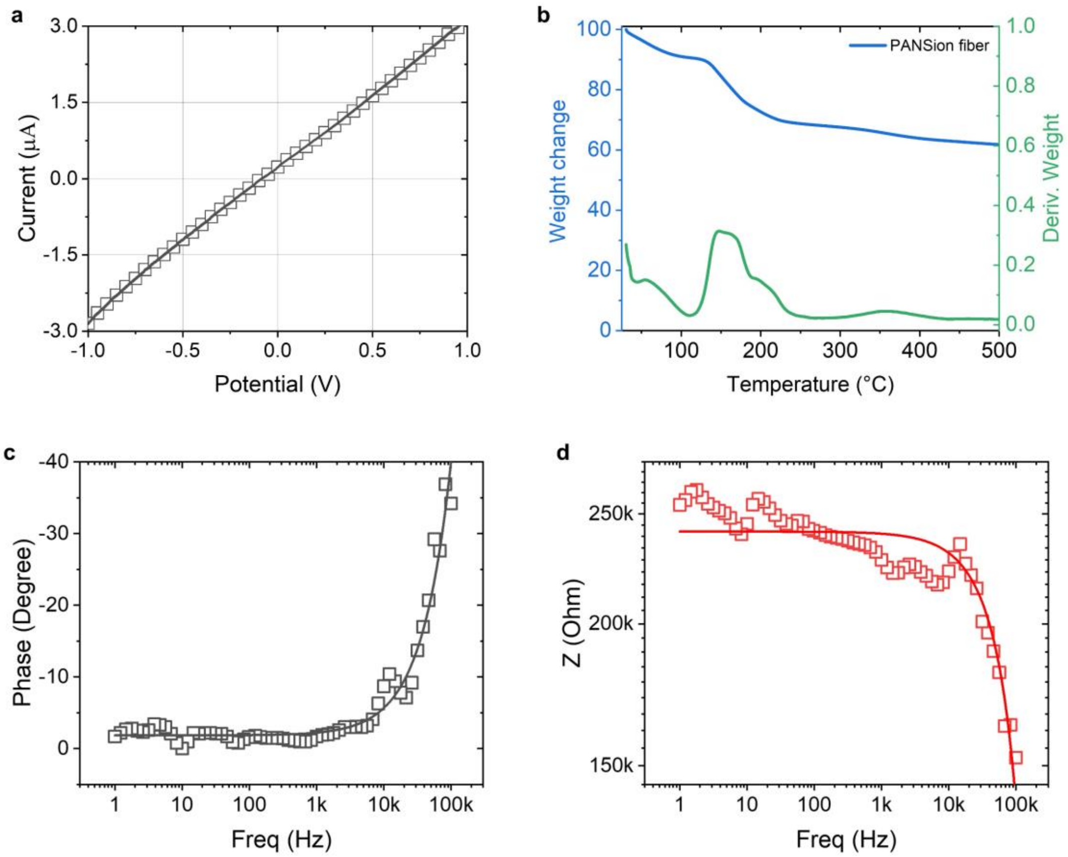

Supplementary Figure 17. Electrical properties of PANSion fibers. a, I-V curve of a PANSion fiber, indicating the ohmic characteristics. **b**, Thermogravimetric analysis showing the residual DMF solvent (~18 wt%). **c–d**, Impedance results of a PANSion fiber: (c) the phase change at different frequencies, and (d) the impedance change as a function of frequency.

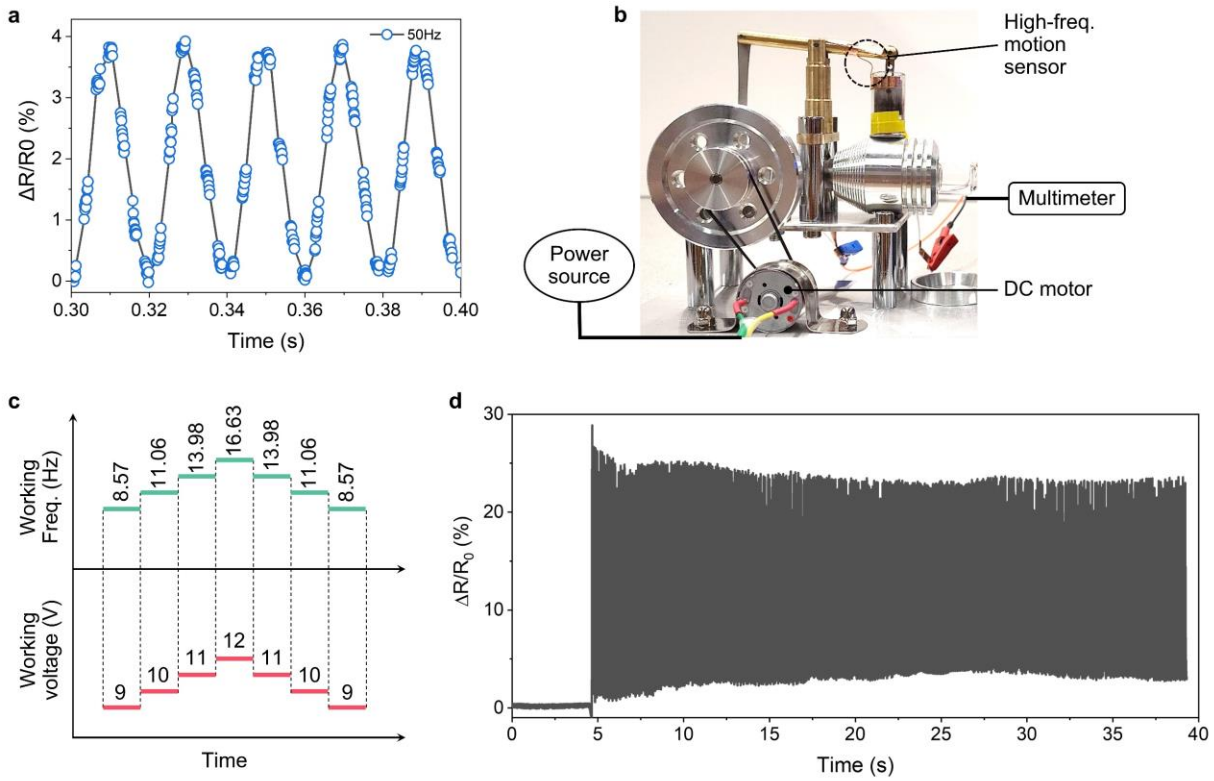

Supplementary Figure 18. Self-sensing fiber for monitoring high-frequency movement. a, Piezoresistive signal of a self-sensing fiber under a 50 Hz stimulus. **b**, Setup of a mechanical-electrical convertor, of which a self-sensing fiber device monitored the high-frequency movement of the lever. **c–d**, The supplied voltage and the corresponding movement frequency of the lever (c) based on the frequency analysis of the collected piezoresistive signals (d).

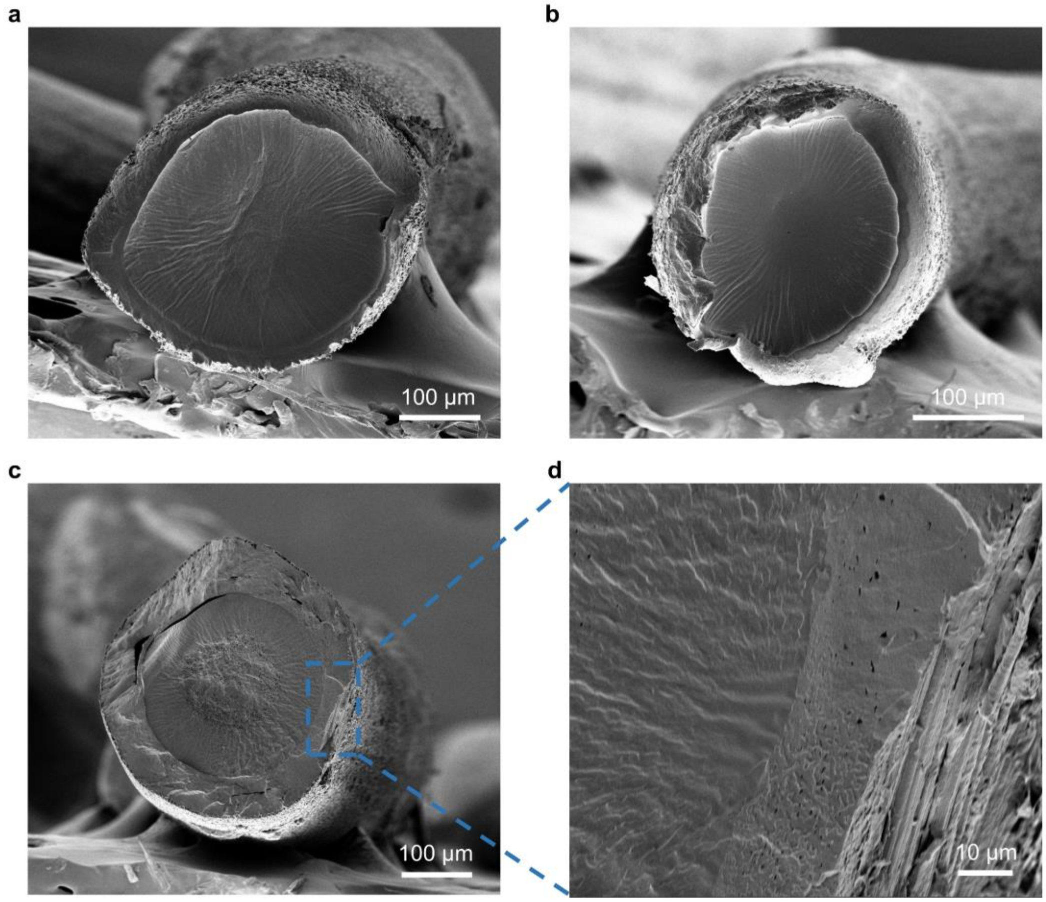

Supplementary Figure 19. Cross-sectional morphology of the TENG fiber using our PANSion fiber as the core and PVDF-HFP as the cortex. a-c, Typical structure of a core-cortex fiber TENG where the PVDF-HFP layer as cortex was evenly coated the PANSion fiber which was the core. **d**, Enlarged view of the interface between the core and cortex.

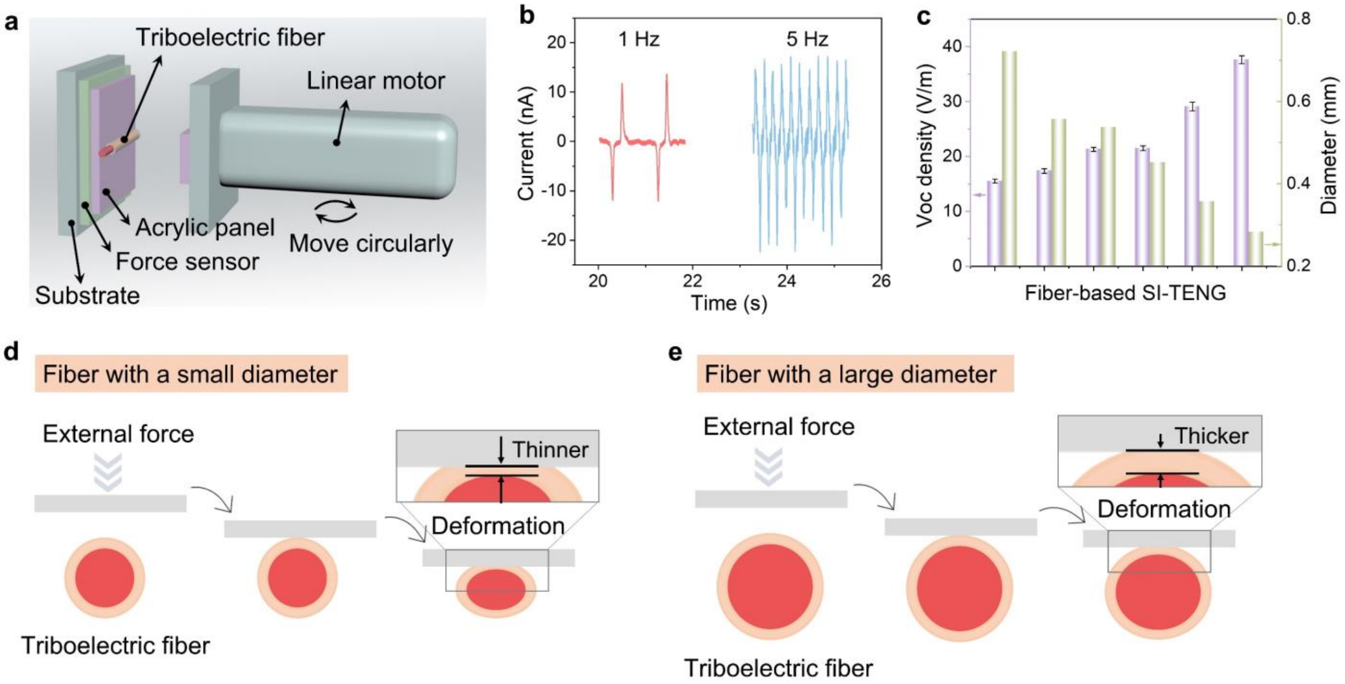

Supplementary Figure 20. Self-powering fiber based on triboelectric mechanism. a, Schematic showing the measurement setup. **b**, Short-circuit current of a triboelectric fiber at 1 Hz and 5 Hz. **c**, Voltage density of a triboelectric fiber with different diameters. **d-e**, Schematic diagram of the deformation for the triboelectric fibers with (d) small diameter and (e) large diameter, respectively, under the same external force. Note that under the same external force, fiber with a small diameter was applied with a high pressure, resulting in a large deformation. Eventually, the cortex layer of fiber with small diameter became thinner than that of fiber with large diameter, which induced more tribo-charges owing to the effective synergism of triboelectrification and electrostatic induction.

Supplementary Table 1. The compositions of PANSion solutions at various molar concentrations of $AgNO_3$ (mol/L).

Dope name	Ratio*	PAN solution (ml)	PAN powder (g)	PAN (mole)	$AgNO_3$ (g)	$AgNO_3$ (mole)	$AgNO_3$ (mol/L)
R2	2	10	1	0.01887	2	0.01177	1.177
R3	3	10	1	0.01887	3	0.01767	1.767
R4	4	10	1	0.01887	4	0.02354	2.354

*The ratio value indicates the solid content between $AgNO_3$ and PAN.

Supplementary Table 2. The comparisons of load resistance for fiber shaped TENGs with coaxial structure.

Fiber TENGs with coaxial structure(core electrode+ sheath fraction layer)	Fiber diameter (mm)	Power density		Load resistance (MΩ)	Refs.
		μW/m	mW/m^2		
Ag NWs/CNTs+PDMS	0.63	21.5	-	150	[6]
PEI/MWCNTs/PA+PEO/WPU/alliin	1.71	2.258×10^3	-	40	[4]
PA@AgNWs yarn+PU	-	0.15	-	10	[8]
Spiral steel wire+silicone rubber	~2.96	2.13	-	100	[9]
Silver-plated nylon yarn+PAN/PVDF nanofiber	0.25	336.2	-	500	[7]
Carbon nanotube yarn+PVDF fibers	0.85	0.072	-	400	[10]
Ag yarn+cotton staple fibers	-	-	213	50	[11]
Ti/Cu-coated nylon film@ZnO+PDMS	-	-	42.6	250	[12]
Stainless-steel fibers+ dielectric fibers	-	-	60	200	[13]
PANSion+PVDF-co-HFP	0.53	0.82	1.64	0.4	This work

References

1. Qin Z, Yin Y, Zhang W, Li C, Pan K. Wearable and Stretchable Triboelectric Nanogenerator Based on Crumpled Nanofibrous Membranes. *ACS Appl Mater Interfaces* **11**, 12452-12459 (2019).
2. Pakawanit P, Pharino U, Charoonsuk T, Sriphan S, Pongampai S, Vittayakorn N. Simple Fabrication of Porous 3D Substrate Polydimethylsiloxane (PDMS) Composited with Polyvinylidene Fluoride-co-Hexafluoropropylene (PVDF-HFP) for Triboelectric Nanogenerator. *Integr Ferroelectr* **222**, 1-13 (2021).
3. Kang G-d, Cao Y-m. Application and modification of poly(vinylidene fluoride) (PVDF) membranes – A review. *J Membr Sci* **463**, 145-165 (2014).
4. Bai Z*, et al.* Constructing highly tribopositive elastic yarn through interfacial design and assembly for efficient energy harvesting and human-interactive sensing. *Nano Energy* **94**, 106956 (2022).
5. Zeng X*, et al.* Fabrication of superhydrophilic PVDF membranes by one-step modification with eco-friendly phytic acid and polyethyleneimine complex for oil-in-water emulsions separation. *Chemosphere* **264**, 128395 (2021).
6. Ning C*, et al.* Flexible and Stretchable Fiber-Shaped Triboelectric Nanogenerators for Biomechanical Monitoring and Human-Interactive Sensing. *Adv Funct Mater* **31**, 2006679 (2021).
7. Ma L*, et al.* Continuous and Scalable Manufacture of Hybridized Nano-Micro Triboelectric Yarns for Energy Harvesting and Signal Sensing. *ACS Nano* **14**, 4716-4726 (2020).
8. Chen J, Wen X, Liu X, Cao J, Ding Z, Du Z. Flexible hierarchical helical yarn with broad strain range for self-powered motion signal monitoring and human-machine interactive. *Nano Energy* **80**, 105446 (2021).
9. Xie L*, et al.* Spiral Steel Wire Based Fiber-Shaped Stretchable and Tailorable Triboelectric Nanogenerator for Wearable Power Source and Active Gesture Sensor. *Nano-Micro Lett* **11**, 39 (2019).
10. Busolo T, Szewczyk PK, Nair M, Stachewicz U, Kar-Narayan S. Triboelectric Yarns with Electrospun Functional Polymer Coatings for Highly Durable and Washable Smart Textile Applications. *ACS Appl Mater Interfaces* **13**, 16876-16886 (2021).
11. Yang Y, Xu B, Gao Y, Li M. Conductive Composite Fiber with Customizable Functionalities for Energy Harvesting and Electronic Textiles. *ACS Appl Mater Interfaces* **13**, 49927-49935 (2021).
12. Li X*, et al.* 3D fiber-based hybrid nanogenerator for energy harvesting and as a self-powered pressure sensor. *ACS Nano* **8**, 10674-10681 (2014).
13. Yu A*, et al.* Core-Shell-Yarn-Based Triboelectric Nanogenerator Textiles as Power Cloths. *ACS Nano* **11**, 12764-12771 (2017).
