# Supplementary material for: Ambient-conditions spinning of functional soft fibers via engineering molecular chain networks and phase separation
Source: Nat Commun. 2023 Jun 5;14:3245. doi: 10.1038/s41467-023-38269-z (PMC10241934; doi:10.1038/s41467-023-38269-z)
Supplement: Supplementary file 2 — Description of Additional Supplementary Files [file 41467_2023_38269_MOESM2_ESM.pdf]

Description of Additional Supplementary Files

File Name: Supplementary Movie 1
Description: Continuous fiber spinning via NVIPS spinning approach using an extrusion-based process

File Name: Supplementary Movie 2
Description: Manual fiber spinning via NVIPS spinning approach. Solvents were removed due to the NVIPS and gravity effects

File Name: Supplementary Movie 3
Description: Manual fiber spinning via NVIPS spinning approach. Solvents were removed due to the NVIPS and gravity effects

File Name: Supplementary Movie 4
Description: Observation of the liquid droplets moving and merging under an optical microscope

File Name: Supplementary Movie 5
Description: Comparison of mechanical properties between fibers prepared via NVIPS spinning and wet spinning approaches. PAN fibers@WS were very brittle. PANSion fibers@WS were not stretchable. PANSion fibers@NVIPS were stretchable and strong

File Name: Supplementary Movie 6
Description: Monitoring high-frequency movement by a selfsensing PANSion fiber
